# Logical intuitions or matching heuristics? Examining the effect of deduction training on belief-based reasoning judgments

**DOI:** 10.3758/s13421-025-01710-3

**Published:** 2025-04-11

**Authors:** Omid Ghasemi, Simon J. Handley, Rachel G. Stephens

**Affiliations:** 1 School of Psychology, University of New South Wales, Sydney, NSW 2052 Australia; 2 School of Psychological Sciences, Macquarie University, Sydney, Australia; 3 School of Psychology, University of Adelaide, Adelaide, Australia

**Keywords:** Dual process, Intuitive logic, Logic training, Matching heuristic, Nonexclusivity, Deductive reasoning

## Abstract

When individuals are asked to evaluate the believability of the conclusions to valid or invalid arguments, they often endorse valid conclusions at higher rates than invalid ones. This effect of validity on belief judgments—the “logic-belief effect” —is considered evidence of intuitive logic. However, recent studies challenged this interpretation by demonstrating that “pseudo-logical” arguments interfere with belief judgments in the same way as valid logical structures do. This finding suggests that a simple heuristic that relies on the matching of constituent propositions, rather than sensitivity to logical validity per se, may lead to the logic-belief effect. To further test this matching heuristic account, across two experiments, we instructed participants to evaluate the conclusions of a series of logical and pseudo-logical arguments based on logic or belief, before and after a logic training block. The results showed that whilst both judgment types were impacted by both logical and pseudo-logical structures before training, after training the effect of the latter was indeed minimized in logic judgments but not belief judgments. The results largely support the matching heuristic account, which have important implications for contemporary dual-process theories of reasoning.

**Supplementary Information:**

The online version contains supplementary material available at 10.3758/s13421-025-01710-3.

## Introduction

Recent research has shown that individuals have a surprising capacity to evaluate the logical validity of an argument under experimental conditions thought to foster more intuitive, fast thinking. For example, people are sensitive to validity even if an experiment’s task simply asks people to assess the believability of an argument’s conclusion (the “logic-belief effect”), how much they like it (the “logic-liking effect”), or how physically bright it is (the “logic-brightness effect”; Ghasemi et al., 2021; Handley et al., 2011; Morsanyi & Handley, 2012; Trippas et al., 2016). Such effects have been seen as evidence for “logical intuitions” or “intuitive logic,” which is important because such intuitions contradict traditional and lay theories of human reasoning which assume that intuitive processes have limited or no access to logic or other normative rules (Morewedge & Kahneman, 2010; Sloman, 1996).

De Neys (2022) has revisited the influential dual-process theory of reasoning in light of these empirical findings, among other findings, proposing the *nonexclusivity* assumption in which intuitive processes can produce normative responses that were once thought to be exclusively the domain of deliberative reasoning. This means that not only deliberative processes, but also intuitive processes can yield logic- and probability-based responses. The assumption of nonexclusivity is important for understanding the interplay between deliberative and intuitive modes of thinking and the switching between them that occurs in various contexts (see De Neys, 2022).

However, despite theoretical and empirical developments in exploring the frequency and extent of logical intuitions (De Neys, 2012; De Neys & Pennycook, 2019; Handley & Trippas, 2015; Newman et al., 2017), such an intuitive grasp of normative rules has been called into question (Ghasemi et al., 2022a, 2023; Handley et al., 2023; Hayes et al., 2020, 2022; Meyer-Grant et al., 2022); in particular, in the case of deductive reasoning tasks, more recent observations suggest that the apparent intuitive logic effects may not be driven by an assessment of the logical structure of an argument. Instead, people may use simple heuristics based on cues that are often confounded with validity—such as whether elements of the conclusion syntactically “match” the premises (Ghasemi et al., 2022a, 2023; Meyer-Grant et al., 2022, 2024). These findings challenge the nonexclusivity assumption of recent dual-process theories, suggesting that intuitive processes, whilst capable of generating responses that are aligned with normative principles, are typically grounded in fast and frugal heuristics rather than an assessment of logic.

Given the theoretical importance of the logic-belief effect, the current study aims to further test the role of a matching heuristic, using stimuli that help to disentangle logical validity and matching cues (Ghasemi et al., 2022a). Crucially, we also explore whether the logic-belief effect is maintained even after participants have been trained to better distinguish valid and invalid arguments, which would strengthen support for the use of a matching heuristic in belief ratings.

### Intuitive logic versus matching heuristics

Traditional dual-process theories of reasoning distinguish between two qualitatively different types of processes (Evans & Stanovich, 2013; Sloman, 1996; but see, Stephens et al., 2018, 2019). Intuitive Type 1 processes are assumed to be the default, providing fast and automatic initial responses to reasoning problems. Deliberative Type 2 processes, on the other hand, are considered to be a slower type of thinking that, depending on the availability of cognitive resources, can override the initial outputs of Type 1 processes. According to these theories, whilst reasoners may be able to produce the correct response by relying on intuitive processes, access and sensitivity to logical, probabilistic, and causal rules are exclusively attributed to deliberative Type 2 processes (Morewedge & Kahneman, 2010; Sloman, 1996).

The strong assumption that intuitive processes lack sensitivity to normative rules has been challenged by studies showing that reasoners produce normatively correct responses under constraints to working memory or time (Bago & De Neys, 2017), feel an intuitive sense of conflict even when they fail to provide the correct answer (Frey et al., 2017; Pennycook et al., 2015), and continue to be influenced by logical validity when they are asked to simply judge the likability, brightness, or believability of arguments (Ghasemi et al., 2021; Morsanyi & Handley, 2012; Trippas et al., 2017). These findings arguably suggest that individuals possess a capacity to engage in rule-based reasoning even under task conditions that foster intuitive processing.

One key approach thought to provide strong evidence in support of the existence of intuitive logic is the *instructional manipulation* paradigm (Handley et al., 2011). In this paradigm, participants are presented with a series of reasoning arguments and are instructed to rate either the believability or logical validity of each argument’s conclusion. Two types of arguments are usually used in such studies. For nonconflict arguments, the validity and prior believability of the argument’s conclusion are congruent (i.e., valid-believable or invalid-unbelievable problems). In conflict arguments, however, validity and believability are incongruent (i.e., valid-unbelievable or invalid-believable problems). Consider the following modus ponens (MP) arguments, which illustrate nonconflict and conflict problems, respectively:

- Argument **A**: MP—valid and believable
- If the tumor disappears (p) then the cancer is cured (q)
- The tumor disappears (p)
- The cancer is cured (q)
- Argument **B**: MP*—invalid and believable
- If the tumor disappears (p) then the cancer progresses (q)
- The tumor disappears (p)
- The cancer is cured (¬q)

When people evaluate arguments like these under logic or belief instructions, different aspects of reasoning are thought to be targeted and different responses are considered normative. Under logic instructions, the extent to which people endorse valid arguments (e.g., Argument A) and reject invalid ones (e.g., Argument B), regardless of the conflict (or believability) of arguments, provides a measure of explicit reasoning. In other words, such a validity effect under logic instructions indicates how well a reasoner can engage in logical reasoning without being influenced by nonrelevant cues to logic such as the believability of the argument, whereas an effect of conflict or believability indicates the extent to which the reasoner is biased. In contrast, when individuals are asked to ignore logical validity and evaluate each argument based on its believability, then they should endorse both Arguments A and B as believable. However, previous research has revealed that people tend to rate Argument A as more believable than Argument B (Handley et al., 2011; Howarth et al., 2016, 2018; Thompson et al., 2018; Trippas et al., 2017). As the researchers have argued, it seems that the logical structures of these arguments are accessible enough to interfere with belief judgments. This interference effect, the logic-belief effect, is considered an indicator of intuitive logic.

Evidence for logical intuitions has led to significant developments in reasoning theory. Mechanisms for the intuitive Type 1 processing of logic have been incorporated into a number of contemporary dual-process accounts, such as the hybrid account (Bago & De Neys, 2020), the parallel processing account (Handley & Trippas, 2015), and the three-stage account (Pennycook et al., 2015). These accounts assume that effects such as the logic-belief, logic-liking, or logic-brightness effects reflect intuitive sensitivity to logical or normative rules. However, there is limited direct evidence to show that access to formal logical rules underlies the effects. It is quite possible, for example, that a more superficial feature that simply often aligns with logical validity, has caused previous observations of the logic-belief, logic-liking, and logic-brightness effects. For example, consider the abstract structure of Arguments A and B above; for the valid MP argument, the p and q elements of the first premise match the p and q propositions in the second premise and conclusion. In contrast, for the invalid MP* argument, there is no such matching as the conclusion asserts ¬q. If a reasoner evaluates these two arguments simply based on a matching heuristic, rather than on more demanding logic rules, she would end up with the same pattern of responses.

Supportive evidence that a matching heuristic drives the logic-belief effect comes from a recent study in which participants were given a series of logical and pseudo-logical arguments, which help to disentangle validity and matching cues (Ghasemi et al., 2022a). For logical arguments, the conclusion either necessarily follows from the premises and hence is valid or determinately true, or it does not follow the premises and hence it is invalid or determinately false (e.g., Arguments A and B above). In contrast, for every pseudo-logical argument, the conclusion does not follow its premises and the argument is logically invalid, but in this case, the conclusion is indeterminate (possibly true but not necessarily so). However, critically, the pseudo-logical arguments vary in their matching cues. Previous deductive reasoning research has shown that individuals tend to regularly endorse the *possible-strong* type of these arguments (e.g., Argument C below) but consistently reject the *possible-weak* type (e.g., Argument D; Evans et al., 2010). In the current study, these arguments will be referred to as *(pseudo-) valid* and *(pseudo-) invalid*, respectively. To illustrate, consider the following affirmation of the consequent (AC) arguments as examples of pseudo-logical problems:

- Argument **C**: AC—pseudo-valid and believable
- If the tumor disappears (p) then the cancer is cured (q)
- The cancer is cured (q)
- The tumor disappeared (p)
- Argument **D**: AC*—pseudo-invalid and believable
- If the tumor grows (p) then the cancer is cured (q)
- The cancer is cured (q)
- The tumor disappeared (¬p)

Note that pseudo-valid arguments like Argument C have a matching between elements, as per the valid MP arguments (i.e., Argument A). In contrast, like the invalid MP argument (i.e., Argument B), such matching is absent for pseudo-invalid arguments like Argument D. Thus, if individuals rely on normative logic rather than a matching heuristic during belief judgments, then the logic-belief effect is expected to be found on logical arguments as indicated by higher endorsement rates of MP than MP* arguments, but AC and AC* endorsements should be similar (i.e., no effect of pseudo-validity). Conversely, if participants evaluate arguments according to the matching of their elements, then higher endorsement of MP and AC compared with MP* and AC* is expected. The results of Ghasemi et al., (2022a) revealed the latter pattern, supporting the matching heuristic hypothesis. More recent research has provided additional evidence that logical intuitions rely primarily on superficial cues, such as the matching heuristic (Ghasemi et al., 2023) and atmosphere effect (Meyer-Grant et al., 2022, 2024), rather than on formal logic.

Together, these findings indicate that apparent evidence for logical intuitions can simply arise from people exploiting superficial features of an argument such as the syntactic matching of its elements, rather than evaluating its underlying logical structure. Because of the important implications for contemporary dual-process accounts that have incorporated logical intuitions (e.g., Bago & De Neys, 2020; Handley & Trippas, 2015; Newman et al., 2017; Pennycook et al., 2015), one of the goals of the current study is to examine the replicability of evidence for the matching heuristic account of the logic-belief effect.

### The effect of training on reasoning

Another core aim of the current study is to examine if training in logic affects the logic-belief effect. After all, if logical intuitions drive the effect, then training should reduce the impact of pseudo-logical structures on belief judgments, whereas if a matching heuristic is at play then training will have little impact. Whilst there is contradictory evidence on the effectiveness of training interventions in debiasing reasoning and decision-making (Prowse Turner & Thompson, 2009; Sellier et al., 2019; Stephens et al., 2020), a large body of research has shown that teaching individuals basic logical and mathematical concepts can result in more normative responses. For example, Isler and colleagues (2020) demonstrated the effectiveness of a debiasing training intervention for well-known reasoning biases such as bat-and-ball and base-rate neglect, by simply using a short explanation of such biases. This improvement in performance was attributed to the activation of deliberative thinking. Similarly, a simple intervention in the form of solving a mathematically similar algebraic equation prior to solving the bat-and-ball problem improved participants’ performance (Hoover & Healy, 2017, 2021). The benefits of training not only transferred into real-world situations (Sellier et al., 2019) but also endured several months later (Franiatte et al., 2024; Morewedge et al., 2015).

Training in logic is also shown to increase the understanding of logic rules such as logical necessity (i.e., a conclusion necessarily follows [or does not follow] from its premises and hence it is valid [or invalid]) and logical possibility (i.e., a conclusion is only possible given its premises and hence it is invalid). For example, Evans et al. (1994) found that whilst a simple instruction to consider logical necessity was not successful in reducing reasoning biases, more complex training including a more elaborated explanation of argument structures and a stronger emphasis on the concept of logical necessity improved reasoning accuracy by minimizing, but not eliminating, belief bias. Similarly, training participants in the logical necessity concept using verbal explanations and Venn diagrams has been successful in increasing the accuracy in evaluating logical (i.e., necessary) and pseudo-logical (i.e., possible) syllogisms (Prowse Turner & Thompson, 2009). Finally, Stephens et al. (2020) presented participants with a series of conditional arguments before and after training in logic, which included both instructional information and training trials with immediate feedback. Participants showed a clear improvement in their deductive (and inductive) reasoning after training, with a higher endorsement of valid arguments and a lower endorsement of invalid arguments.

Although most studies attributed the improvement in performance to activation or reinforcement of deliberative reasoning, more recent research has shown that training can boost performance in intuitive task conditions, such as under time pressure. For example, Boissin et al. (2021) asked participants to solve Cognitive Reflection Test (CRT) problems once under time and memory constraints (Time 1) and once with no constraints (Time 2), across pre- and post-intervention phases. A short training intervention was provided to half of the participants, explaining the nature of the problems, while the other half did not receive training. The results revealed that correct responses were mostly produced at Time 1 rather than only at Time 2, and the training further improved their Time 1 responses. Similarly, Purcell et al. (2020) found similar effects, with training boosting CRT accuracy and reducing the impact of working memory load. Altogether, these findings demonstrate that training participants in basic logical and mathematical concepts is successful in improving reasoning in both intuitive and deliberative conditions. The current study will examine whether similar post-training improvements generalize to both logic and belief judgments for logical and pseudo-logical arguments.

It is important to note that we would expect training to boost any logical intuitions for belief judgements because the purpose of such short training interventions is primarily to reinforce and clarify existing logical rules, rather than to automate new logical structures or build mindware (i.e., a set of learned skills in and knowledge of normative rules; Stanovich, 2018). We see the training as helping to trigger relevant stored knowledge and motivate individuals to exploit it. This bootstrapping from prior knowledge is most likely an important reason why even simple debiasing interventions such as providing participants with correct and incorrect answers to a problem along with a short explanation are effective in improving intuitive reasoning (Boissin et al., 2021; Purcell et al., 2020).

### The present study

This study investigates whether the logic-belief effect arises through the rapid activation of a matching heuristic or the intuitive application of logical rules, as assumed by contemporary dual-process accounts (De Neys & Pennycook, 2019; Handley & Trippas, 2015; Newman et al., 2017). There are two primary aims. First, the study aims to replicate effects of both logic and pseudo-logic on belief judgments for untrained reasoners, consistent with the matching heuristic account. Second, the study aims to test whether training in conditional rules has a differential impact on reasoning under logic versus belief instructions.

Under logic instructions, we would expect training to facilitate participants’ capacity to distinguish between logical and pseudo-logical arguments, more often endorsing valid arguments and rejecting invalid arguments (including rejecting all pseudo-logical arguments). The key question is whether the training also has a similar effect on belief judgments, suggesting that people evaluate logic during belief judgments. Alternatively, the absence of a training impact on belief judgments for both logical arguments (valid vs. invalid) and pseudo-logical arguments (pseudo-valid vs. pseudo-invalid) would further support the matching hypothesis. To that end, we conducted two experiments, each with pre-training, training, and post-training blocks. In the pre- and post-training blocks, we asked participants to evaluate a series of logical and pseudo-logical arguments either under logic instructions or belief instructions.

If logic truly drives the logic-belief effect, then a (pseudo-) validity effect under belief instructions is expected on logical, but not pseudo-logical arguments. Thus, the training should eliminate the logic-belief effect on pseudo-logical arguments if the effect is driven by logic. On the other hand, if the logic-belief effect arises because of the rapid activation of a matching heuristic rather than logic, then we expect the (pseudo-) validity effect under belief instructions on both logical and pseudo-logical arguments—even after training.

## Experiment 1

Experiment 1 addressed the two main aims of replicating evidence for the matching heuristic account and further testing this account via examining the effects of logic training. In this experiment, we presented participants with a series of conditional arguments before and after receiving training in deductive logic. Participants were instructed to evaluate each argument’s conclusion based on either its logical validity or believability. Half of the conditionals were logical arguments and the other half were pseudo-logical arguments. Whilst logical arguments were either logically valid or invalid, pseudo-logical arguments were all logically invalid. However, we coded half of the pseudo-logical arguments that are usually endorsed by individuals (i.e., the propositions match) as “(pseudo-) valid” arguments and the other half that are regularly rejected by individuals (i.e., the propositions do not match) as “(pseudo-) invalid” arguments. To investigate the use of a matching heuristic, we examined the “(pseudo-) validity effect” before and after the training, which involved comparing participants’ judgments for valid versus invalid arguments for logical arguments, and comparing judgments for pseudo-valid versus pseudo-invalid arguments for pseudo-logical arguments.

### Method

#### Ethics statement

Both experiments were approved by the Macquarie University Human Science Ethics Committee (Approval Number 26289) and the University of Adelaide Human Research Ethics Subcommittee (Approval Number 21/24). Informed consent was obtained from all participants prior to each experiment.

#### Participants

For this experiment, we recruited 202 undergraduate students (144 women, 55 men, and three nonbinary/trans; *M*_age_ = 22.8, *SD* = 7.74) from Macquarie University (*N* = 136) and the University of Adelaide (*N* = 66). Subjects received course credit to participate in a 50-min experiment.

#### Design and materials

We used a 2 × 2 × 2 × 2 × 2 within-subjects design, manipulating validity (valid vs. invalid), believability (believable vs. unbelievable), argument type (logical vs. pseudo-logical), instruction (logic vs. belief), and block (pre-training vs. post-training). Participants were presented with 64 conditional arguments in total. Half of the arguments were logical arguments which included 16 modus ponens (MP or MP*) and 16 modus tollens (MT or MT*) conditionals, and the other half were pseudo-logical arguments which included 16 affirmation of the consequent (AC or AC*) and 16 denial of the antecedent (DA or DA*) conditionals (see Table 1). Whilst logical arguments were either valid or invalid, all pseudo-logical arguments were invalid as the conclusion does not necessarily follow the premises according to deductive logic rules. However, participants tend to endorse AC and DA arguments as valid (so are usually called possible-strong arguments) and tend to reject AC* and DA* arguments as invalid (so are usually called possible-weak arguments). We labelled the former as (pseudo-) valid and the latter as (pseudo-) invalid in our analysis.

**Table 1:** Examples of logical (MP and MT) and pseudo-logical (AC and DA) arguments across (pseudo-) validity and believability conditions of Experiment 1

Argument Type	Valid/Believable	Valid/Unbelievable	Invalid/Believable	Invalid/Unbelievable
Modus ponens	P1: If John is hungry [p] then he has eaten too little [q] P2: John is hungry [p] C: John has eaten too little [q]	P1: If John is hungry [p] then he has eaten too much [q] P2: John is hungry [p] C: John has eaten too much [q]	P1: If John is hungry [p] then he has eaten too much [q] P2: John is hungry [p] C: John has eaten too little [¬q]	P1: If John is hungry [p] then he has eaten too little [q] P2: John is hungry [p] C: John has eaten too much [¬q]
Modus tollens	P1: If John is hungry [p] then he has eaten too little [q] P2: John has eaten too much [¬q] C: John is full [¬p]	P1: If John is full [p] then he has eaten too little [q] P2: John has eaten too much [¬q] C: John is hungry [¬p]	P1: If John is full [p] then he has eaten too little [q] P2: John has eaten too much [¬q] C: John is full [p]	P1: If John is hungry [p] then he has eaten too little [q] P2: John has eaten too much [¬q] C: John is hungry [p]
	**Pseudo-Valid/Believable**	**Pseudo-Valid/Unbelievable**	**Pseudo-Invalid/Believable**	**Pseudo-Invalid/Unbelievable**
Affirmation of the consequent	P1: If John is hungry [p] then he has eaten too little [q] P2: John has eaten too little [q] C: John is hungry [p]	P1: If John is full [p] then he has eaten too little [q] P2: John has eaten too little [q] C: John is full [p]	P1: If John is full [p] then he has eaten too little [q] P2: John has eaten too little [q] C: John is hungry [¬p]	P1: If John is hungry [p] then he has eaten too little [q] P2: John has eaten too little [q] C: John is full [¬p]
Denial of the antecedent	P1: If John is hungry [p] then he has eaten too little [q] P2: John is full [¬p] C: John has eaten too much [¬q]	P1: If John is hungry [p] then he has eaten too much [q] P2: John is full [¬p] C: John has eaten too little [¬q]	P1: If John is hungry [p] then he has eaten too much [q] P2: John is full [¬p] C: John has eaten too much [q]	P1: If John is hungry [p] then he has eaten too little [q] P2: John is full [¬p] C: John has eaten too little[q]

We also manipulated the believability of arguments, so half of the arguments had a believable conclusion and the other half had an unbelievable conclusion. To construct our materials, we used implicit negations (happy vs. sad) instead of explicit ones (happy vs. not happy). Although some implicit negation categories may not be considered to be binary (e.g., a person may be happy, sad, anxious) and hence, affect the logical validity of some valid MT arguments, previous research has found that participants show a very similar responding pattern for arguments with implicit and explicit negations (Ghasemi et al., 2022a). Moreover, as we used explicit negations in our training materials, to encourage participants to employ their newly learned logic knowledge and not superficial matching heuristics in evaluating arguments, we decided to use implicit negations in creating the pre- and post-training materials.

We presented 32 arguments in each pre- and post-training block. To avoid repeating arguments with the same content in each judgment block, we created two lists of arguments, each with eight unique contents. Thus, each list contained eight arguments for each of MP, MT, AC, and DA structures (2 replicates for each validity × believability condition). For each participant, we randomly assigned each list to either the pre-training block or the post-training block. Moreover, to avoid the confounding effect of content on the validity status of arguments (Klauer & Singmann, 2013), we randomly assigned contents to experimental conditions for each participant. Thus, for example, an argument with the content of “John-Hungry-Full” (as per Table 1) could be a (pseudo-) valid believable item for one participant and a (pseudo-) invalid unbelievable for another participant. All the arguments were adapted from Ghasemi et al., (2022a). An example of each argument type can be found in Table 1 and the full list of all arguments can be retrieved from OSF (https://osf.io/r382w/).

#### Procedure

**Fig. 1:**
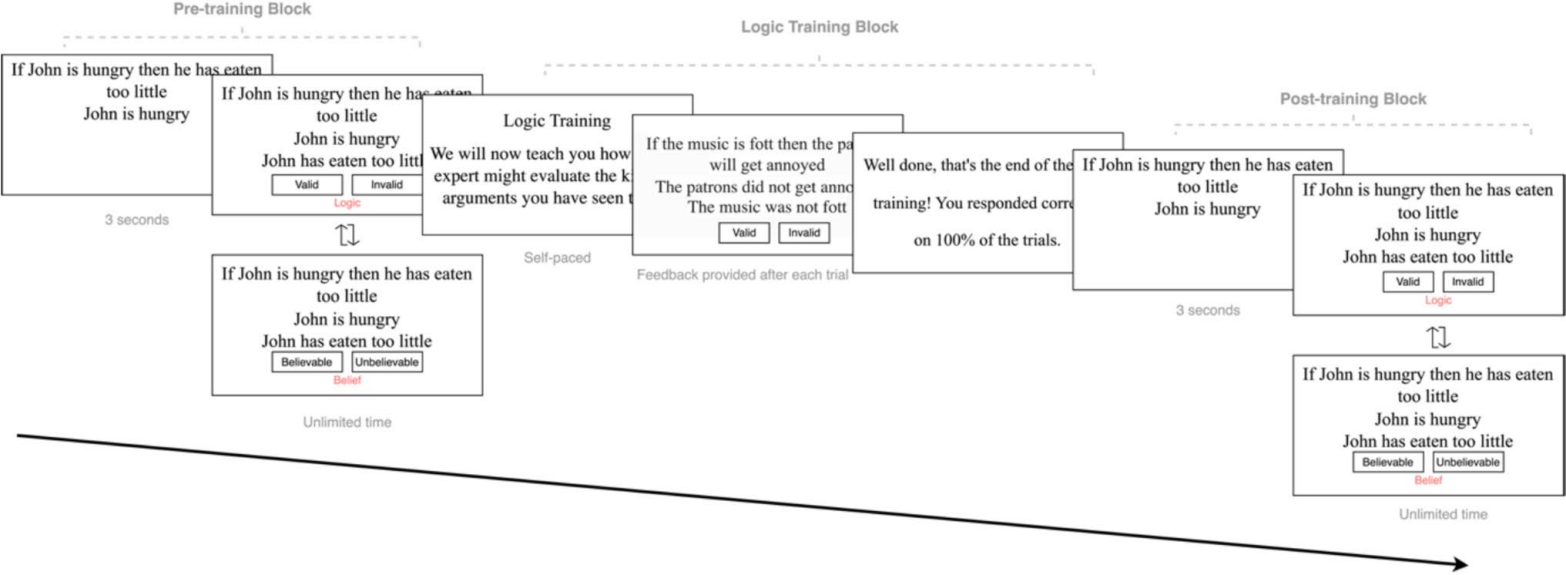
A visual summary of experimental procedure outlining the sequence of tasks and training phases. (Color figure online)

Both Experiments 1 and 2 were conducted online and were created using JsPsych (De Leeuw, 2015). Experiment 1 consisted of a pre-training block, a training block, and a post-training block (see Fig. 1 for an overview of Experiment 1 procedure). In the pre- and post-training blocks, participants were presented with 64 arguments in total and were asked to evaluate each item either based on logical validity or believability. Under logic instructions, participants were instructed to assume the two premises are true and to judge whether the conclusion necessarily follows from those premises. Under belief instructions, participants were instructed to evaluate each argument based on the congruency of the conclusion to their general knowledge of the world.

We randomized the order of presentation of items for each participant in each block, with logic/belief instructions randomly interleaved across trials. We used a serial presentation format in which both premises were initially presented on the screen, and after 3 s the conclusion appeared along with response options and an instructional cue in red color. On each trial, if the cue was “Logic,” the response options were “Valid” and “Invalid,” and if the cue was “Belief,” then the options were “Believable” and “Unbelievable.” Instructional cues were randomly assigned to the other experimental conditions so that the design remained fully counterbalanced. After selecting an option, participants were asked to rate their confidence in their response on a 6-point scale from 50% (guessing) to 100% (certain). We only focused on the endorsement judgments and confidence ratings data were not analyzed.

After the pre-training block, participants were presented with a logic training block which was adapted from Stephens et al. (2020). The training was created to teach participants how to approach and evaluate conditional arguments as “logicians” do. Thus, they were presented with the abstract forms of four conditional arguments and, using Euler diagrams, were trained on the reasons why MP and MT arguments are valid and AC and DA arguments are logically invalid. To be clear, in order to simplify training materials by focusing on only four key structures, we trained participants with the valid and pseudo-valid structures (i.e., the two leftmost columns of Table 1), but not with invalid (MP* and MT*) and pseudo-invalid (AC* and DA*) structures. Prior to the training, participants were instructed to read the training materials carefully as there would be a short logic test after the training. This test was included to enhance participants’ engagement with the materials and assess the effectiveness of the training. The training materials were presented as a series of self-paced slides that participants could navigate within and return to previous slides. Finally, at the end of the block, participants were asked to judge the validity of 12 test arguments; three arguments for each of the trained conditional structures and were given immediate feedback in the form of “Correct” and “Incorrect” for their response to each item. These items did not vary in believability via the inclusion of a nonsense word (e.g., If the music is fott then the patrons will get annoyed; The music was fott; The patrons got annoyed). After evaluating these training test items, participants were presented with a summary of their results, including their accuracy in total and for each argument type separately. Finally, participants proceeded to the post-training block and were reminded of the instructions to evaluate arguments according to the belief or logic cues.

### Results

#### Analysis approach

We used a Bayesian hierarchical logistic regression analysis (i.e., the Bayesian generalized mixed-model analysis) to test our hypotheses. Our analysis was hierarchical as it included both fixed and random effects to take into account the variability of participants and items. For the random-effect structure, we ran the maximal model including by-participants and by-item random intercepts and random slopes for all fixed effects, as well as a correlation parameter. Such a maximal model is recommended to guard against Type 1 error (Barr et al., 2013). We also used a Bernoulli distribution with a logit link function to model the binary endorsement rates data and hence, our analysis was a logistic regression. Finally, we used a Bayesian parameter estimation approach (Kruschke, 2014; Kurz, 2021) to determine the credibility of an effect. Following this approach, we extracted the posterior distribution for each effect along with its credible intervals. For the Bayesian credible intervals, we used the highest density interval (HDI) which is the interval that includes 95% of credible values. If the null value (in our case, zero) lies outside the HDI of distribution, we consider that effect as credible. Otherwise, this effect is considered as noncredible. For more important effects, we plotted posterior distributions so readers can judge the [non-]credibility of each effect beyond the binary decision rule. Moreover, as it is common to define a region of practical equivalence (ROPE) around the null value, posterior distributions can be used to directly examine if a HDI fully excludes the ROPE or not. Whilst HDI values can be estimated by and extracted from the models, ROPE is usually defined theoretically, based on expert opinions or previous research.

Besides the parameter estimation approach, we used Bayesian model comparison as a further test of the key effects of the study. We used the leave-one-out cross-validation index (“loo”; Vehtari et al., 2017) to compare models with and without a term. A model that has a better accuracy in predicting out-of-sample data would have a smaller information criterion (i.e., loo). This approach allows us to test if adding a term (e.g., an interaction term) would increase the predictive accuracy of a model, and hence, if that term can be considered reliable.

For each experiment, we performed two Bayesian models, one for each instruction condition. Thus, we modeled endorsement rates data with (pseudo-) validity (valid vs. invalid), belief (believable vs. unbelievable), argument type (logical vs. pseudo-logical), and block (pre-training vs. post-training) as zero-sum coded fixed effects and all possible random effects. As we used a zero-sum coded contrast and a logit link, the model estimate was half the differences of the levels of a predictor on a log odd scale. We reported this estimate as the posterior mean of an effect (i.e., M_post_). However, for simplifying the interpretation, we also reported the posterior mean of the differences (e.g., valid − invalid) on the original scale by converting the log odd scale to the probability scale (i.e., M_diff_). The difference of differences (e.g., valid − invalid in pre-training − post-training blocks) are also reported as M_dod_. As already explained, although logical arguments were either valid or invalid, all pseudo-logical arguments were inherently invalid. However, following Ghasemi et al., (2022a), we coded possible-strong arguments as valid and possible-weak ones as invalid. Thus, we refer to the validity factor in our design as “(pseudo-) validity.”

Finally, we used weakly-informative priors so that the posteriors were mostly informed by the data. For each model, we used 4 chains, each with 5000 iterations and 1000 warmups, and hence, we had 16,000 postwarmup draws. Model structures, prior specifications, diagnostics, and posterior predictive checks can be found in the supplementary Materials.

#### Logic training test performance

The results of the 12 training test trials showed that participants had an overall accuracy of 67.1% (*SD* = 20.9), with 90.3%, 72.1%, 54%, and 52% accuracies for MP, MT, AC, and DA arguments, respectively. The overall accuracy was lower than the 78% and 82% accuracy levels in Experiments 1 and 2 of the Stephens et al.’ (2020) study.

#### Logic judgments

This section reports the results of a Bayesian hierarchical logistic regression of logic instruction trials during pre- and post-training blocks. The results of the model revealed credible main effects of (pseudo-) validity (M_post_ = 2.02, HDI [1.80, 2.25]), belief (M_post_ = 0.82, HDI [0.65, 1]), and block (M_post_ = 0.28, HDI [0.16, 0.40]). (Pseudo-) valid arguments were endorsed more than (pseudo-) invalid arguments (M_diff_ = 0.75, HDI [0.70, 0.79]), and believable arguments were endorsed more than unbelievable ones (M_diff_ = 0.27, HDI [0.30, 0.44]). Participants endorsed more arguments during pre-training than post-training (M_diff_ = 0.13, HDI [0.07, 0.19]).

**Fig. 2:**
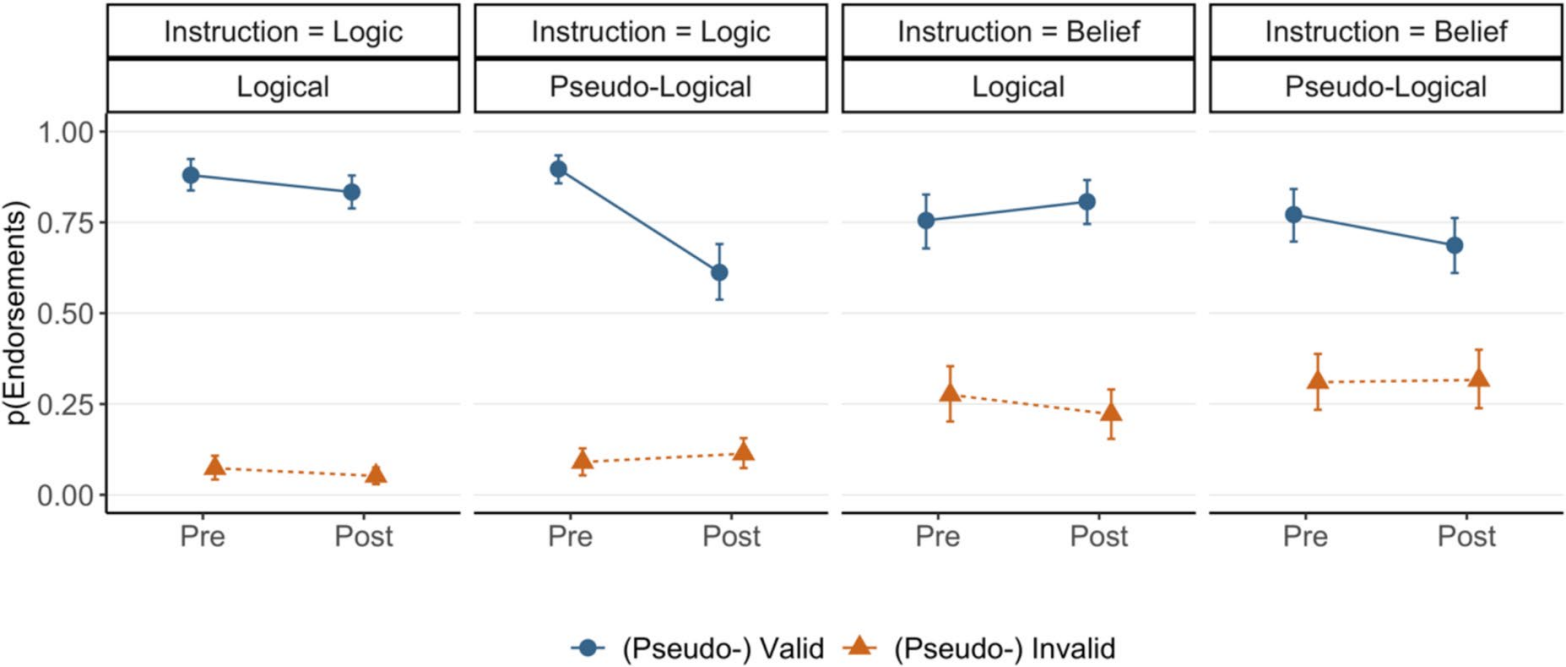
Estimated endorsement ratings of (pseudo) valid and (pseudo-) invalid logical and pseudo-logical arguments under belief and logic instructions across training blocks of Experiment 1. Error bars represent 95% highest density intervals

The results showed that (pseudo-) validity interacted with block (M_post_ = 0.25, HDI [0.06, 0.45]). The (pseudo-) validity effect (i.e., valid—invalid or pseudo-valid—pseudo-invalid) was found to be larger in the pre-training block (M_diff_ = 0.81, HDI [0.75, 0.86]) than the post-training block (M_diff_ = 0.66, HDI [0.59, 0.73]). Such a reduction is the result of a lower endorsement rate for pseudo-valid arguments after logic training (explored further below). We also found a (pseudo-) validity by argument type interaction (M_post_ = 0.26, HDI [0.14, 0.38]) which indicates a more pronounced (pseudo-) validity effect on logical (M_diff_ = 0.80, HDI [0.75, 0.84]) than pseudo-logical arguments (M_diff_ = 0.69, HDI [0.63, 0.75]). Crucially, this effect was modified by a higher-order interaction with block (M_post_ = 0.24, HDI [0.13, 0.36]). Visual inspection of Fig. 2 suggests that, in the pre-training block, the (pseudo-) validity effects are similar for logical (M_diff_ = 0.81, HDI [0.74, 0.87]) and pseudo-logical (M_diff_ = 0.81, HDI [0.74, 0.87]) arguments. However, in the post-training block, participants endorsed pseudo-logical arguments less often which resulted in a smaller (pseudo-) validity effect for pseudo-logical arguments (M_diff_ = 0.50, HDI [0.41, 0.60]) than logical ones (M_diff_ = 0.78, HDI [0.72, 0.84]). It seems that the training was successful in suppressing the endorsement of pseudo-valid arguments for logic judgments. We also found a reliable belief by block interaction (M_post_ = 0.16, HDI [0.03, 0.30]). No other effects were found to be credible. Additional figures illustrating all experimental conditions, including believability, are provided in the Supplementary Materials.

#### Belief judgments

For the trials with belief instructions during pre- and post-training blocks, we performed the same Bayesian analysis on endorsement judgments. The results demonstrated credible main effects of (pseudo-) validity (M_post_ = 1.05, HDI [0.86, 1.25]) and belief (M_post_ = 2.29, HDI [2.01, 2.58]); participants endorsed (pseudo-) valid arguments more than (pseudo-) invalid ones (M_diff_ = 0.48, HDI [0.41, 0.55]) which demonstrates a reliable logic-belief effect, consistent with the matching heuristic account. Participants also endorsed believable arguments more than unbelievable ones (M_diff_ = 0.81, HDI [0.77, 0.86]). As expected, under belief instructions compared with logic instructions, a smaller yet credible (pseudo-) validity effect, and a larger belief effect were found.

As for the logic judgments, we found a (pseudo-) validity by argument type interaction (M_post_ = 0.15, HDI [0.03, 0.28]) which indicates a larger (pseudo-) validity effect (i.e., valid – invalid or pseudo-valid – pseudo-invalid) on logical (M_diff_ = 0.54, HDI [0.45, 0.62]) than pseudo-logical arguments (M_diff_ = 0.42, HDI [0.33, 0.51]). As Fig. 2 suggests, the smaller (pseudo-) validity effect on pseudo-logical arguments is mainly the result of training as participants endorsed pseudo-valid arguments less often and valid arguments more often in the post-training block compared with the pre-training block. This pattern was supported by a three-way interaction of (pseudo-) validity, argument type, and block (M_post_ = 0.13, HDI [0.03, 0.24]). Whilst in the pre-training block, the (pseudo-) validity effects on logical (M_diff_ = 0.48, HDI [0.37, 0.59]) and pseudo-logical (M_diff_ = 0.46, HDI [0.35, 0.57]) arguments were similar, in post-training, this effect increased for logical arguments (M_diff_ = 0.59, HDI [0.49, 0.68]) and decreased for pseudo-logical arguments (M_diff_ = 0.37, HDI [0.25, 0.49]). However, the HDIs of the last two interactions barely exclude zero as a credible value, and thus, according to the parameter estimation approach, these effects should be considered with caution as a reasonable ROPE around zero can include some values of HDI (Kruschke, 2014).

Using a model comparison approach to pitting models with and without the (pseudo-) validity by argument type by block interaction against each other, we found that the model without the three-way interaction term (elpd loo =  − 4205.79, *SE* = 22.84) had a similar out-of-sample predictive accuracy to the model with this term (elpd loo =  − 4207.69, *SE* = 22.91) as the error bars of the models overlap. Therefore, including the three-way interaction in the model does not improve the predictive accuracy of the model and such an effect can be considered as not reliable. The plot of the predictive accuracy of models for both Experiments 1 and 2 can be found in the Supplementary Materials. Finally, we found credible (pseudo-) validity by belief (M_post_ = 0.13, HDI [0.02, 0.25]) and also belief by block (M_post_ = 0.16, HDI [0.02, 0.31]) interactions. No other effects were credible.

**Fig. 3:**
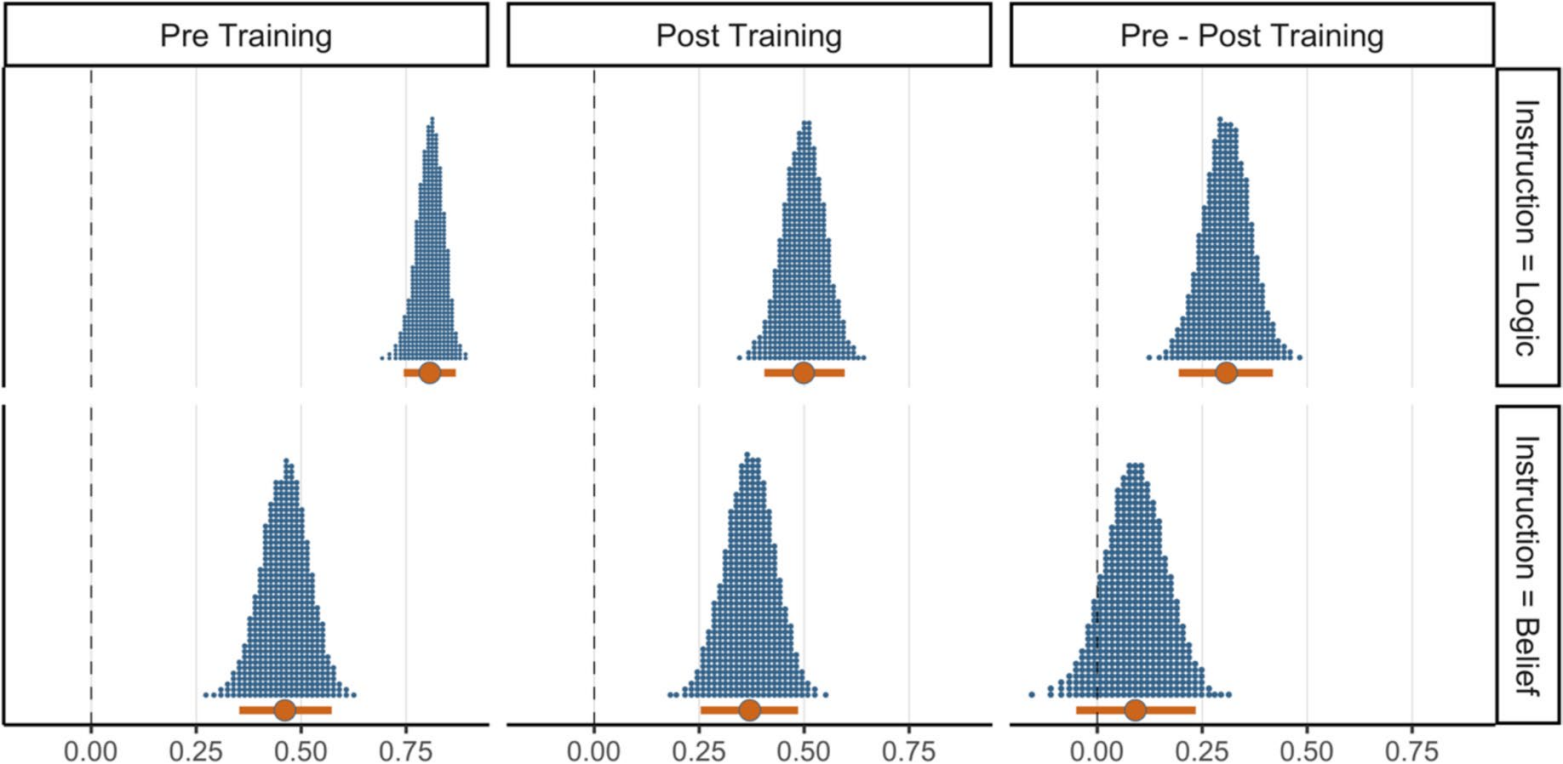
The distributions of validity effects for pseudo-logical arguments under logic and belief instructions of Experiment 1 in each training block along with their contrast. The central circles represent posterior means of the effects and error bars represent 95% HDIs

To further test the competing logical intuitions and matching heuristic accounts, we examined the impact of training on logic and belief judgments for pseudo-logical arguments. We had predicted that if logic evaluation contributes to both kinds of judgments, logic training should reduce the impact of pseudo-logical structures on both belief and logic judgments. Alternatively, if a matching heuristic is behind the logic-belief effect, logic training may selectively improve logic judgments (i.e., reduce the effect of pseudo-validity) yet not affect belief judgments. Figure 3 highlights the relevant results, depicting the posterior predictive draws of the pseudo-validity effect on judgments for pseudo-logical arguments, across training blocks and instructions. Differences between training blocks are shown in the rightmost column. The figure supports the matching heuristic account; logic training reduced the pseudo-validity effect for logic judgments (M_dod_ = 0.31, HDI [0.19, 0.42]). However, under belief instructions, whilst the pseudo-validity effect was smaller during post-training compared with pre-training, it was still credible (M_diff_ = 0.37, HDI [0.25, 0.49]) and 100% of the posterior draws were still above the null value of zero. More importantly, the difference in the pseudo-validity effect between the training blocks was not credible as the HDI of the difference distribution (the rightmost column of the second row) includes zero as a credible value (M_dod_ = 0.09, HDI [− 0.05, 0.23]). These results indicate that whilst the logic training was an effective intervention in improving performance under logic instructions, it had a minimal effect on the impact of pseudo-validity on belief judgments. This selective training effect suggests that the logic-belief effect is not driven by the activation of normative rules (which is facilitated by training) but is consistent with the operation of a matching heuristic.

### Discussion

In Experiment 1, we tested the “matching heuristic” and logical intuition accounts and examined whether training in logic reduces the effect of matching cues in favor of logic assessment, for both logic and belief judgments. The results showed that for untrained reasoning prior to the training block, we replicated the logic-belief effect for logical arguments; belief judgments were affected by validity (Trippas et al., 2017). Moreover, we replicated previous evidence for the matching heuristic account (Ghasemi et al., 2022a, 2023); belief judgments were similarly affected by pseudo-validity. Participants endorsed pseudo-valid arguments as much as true valid arguments and rejected pseudo-invalid arguments as much as true invalid arguments. This suggests that participants were considering the matching of propositions during belief judgments, rather than assessing logical structure.

The results also showed that logic training was somewhat effective in reducing logical errors in logic judgments. In particular, after receiving training in deductive logic, participants started to reject pseudo-valid arguments when they were instructed to evaluate the logical validity of those arguments. This finding is in line with studies showing the effectiveness of a simple training intervention in improving logic judgments (Prowse Turner & Thompson, 2009; Stephens et al., 2020) and indicates that the logic training in this experiment is a reasonably successful intervention to boost reasoning accuracy.

The key question we addressed was whether the effectiveness of logic training on logic judgments translated to a reduced endorsement of pseudo-logical arguments under belief judgments, which would support that belief judgments are affected by logical intuitions rather than a matching heuristic. Before training, belief judgments were heavily influenced by pseudo-validity, and training had little impact on removing this effect. The finding is consistent with the idea that the effects of both validity and pseudo-validity on belief judgments are predominantly driven by a matching heuristic. The limited impact of training on belief judgments for pseudo-logical arguments does not support the logical intuitions account, according to which, logic assessment drives the logic-belief effect.

One might argue that the training in Experiment 1 did not sufficiently affect belief judgments because it had three major limitations. First, perhaps some of the logical structures we used were too complex. As recent theoretical and empirical studies have claimed, intuitive logic is primarily the result of an automatization process in which simple logic rules had been trained and practiced enough so that individuals can automatically and intuitively use or evaluate them (Boissin et al., 2021; De Neys & Pennycook, 2019; Handley & Trippas, 2015). An example of such simple logic rules is the MP conditional: “*If I study hard for the exam (p), then I will pass (q); I studied hard in the last few days (p); Therefore, I will pass the exam today (q).*” In our training materials, however, we used more complex conditional forms such as MT and DA structures that are found to be among the hardest reasoning arguments (Ricco et al., 2020). Several studies have shown that individuals may lack an intuitive sensitivity to such complex logical rules (Brisson et al., 2018; Trippas et al., 2017). Thus, it is possible that our training did not affect belief judgments because there was insufficient preexisting mindware to be activated.

Second, to keep the training relatively simple and short, we trained participants only in (pseudo-) valid forms but not in (pseudo-) invalid ones. Perhaps this training procedure meant that participants were not sufficiently equipped to distinguish logical and pseudo-logical structures. The results revealed a limited, if not zero, carry-over effect to untrained structures, as only a small post-training reduction in the endorsement of (pseudo-) invalid arguments was observed. Thus, if we had taught participants with both (pseudo-) valid and (pseudo-) invalid structures, they might have shown a smaller pseudo-validity effect for pseudo-logical arguments.

Third, due to the online nature of Experiment 1, it is likely that participants did not fully engage in the training material. Such a minimal engagement level is suggested by the training test accuracy of 67%; whilst above the chance level of 50%, it was lower than the training test accuracy of 78% and 82% in Stephens et al. (2020) (which was instead run a more controlled laboratory environment), from which the current training materials were adapted. Thus, encouraging participants to engage more in training could be beneficial in disentangling matching and intuitive logic strategies. In Experiment 2, we modified our training intervention to address these limitations.

## Experiment 2

The results of Experiment 1 revealed that whilst training individuals in deductive logic was successful in enhancing their reasoning accuracy for logic judgments, it had little effect on the impact of (pseudo-) validity for belief judgments. This suggests that when assessing argument believability, people do not also intuitively assess logic but are influenced by other superficial features such as the matching of the conditional propositions. However, as already discussed, the limited impact of training on belief judgments may have arisen from the limitations of our training intervention; specifically, including highly complex logical arguments, excluding the (pseudo-) invalid forms, and failing to motivate participants to engage more carefully.

In Experiment 2, we addressed these limitations by (1) simplifying the set of argument structures used across all blocks, (2) introducing all these structures in the training, including (pseudo-) invalid forms, and (3) implementing procedures to increase active engagement with the training materials. To that end, we used only MP and AC conditionals as logical and pseudo-logical arguments and removed MT and DA structures. Moreover, participants were trained on both (pseudo-) valid and (pseudo-) invalid forms of MP (i.e., MP and MP*) and AC (i.e., AC and AC*) arguments. Finally, at the beginning of the training block, participants were informed that they needed to reach 90% accuracy in the training check test to be able to proceed to the next block, and if accuracy was lower than 90%, they would be returned to the beginning of the training block.

### Method

#### Participants

One hundred and thirty undergrads (93 women, 35 men, one nonbinary/trans, and one other; *M*_age_ = 22.5 years, *SD* = 18.6) from Macquarie University (*N* = 55) and the University of Adelaide (*N* = 75) participated in this experiment. As in Experiment 1, participants received course credit for a 50-min experiment.

#### Materials

As in Experiment 1, participants were presented with 32 arguments in the pre-training block and 32 arguments in the post-training block. However, in Experiment 2, we used only modus ponens (MP) and affirmation of the consequent (AC) conditional arguments as logical and pseudo-logical arguments, respectively. No modus tollens (MT) and denial of antecedent (DA) arguments were used. For both MP and AC conditionals, we factorially crossed (pseudo-) validity and believability. Moreover, similar to Experiment 1, we created two lists of arguments, each with 16 unique contents that were randomly assigned to either the pre- or post-training blocks. We used each of these 16 contents to create one MP argument and one AC argument. Finally, we used the same content randomization and implicit negation implementation as in Experiment 1.

#### Procedure

We used the same procedure as in Experiment 1, according to which participants were presented with a pre-training block, a logic training block, and a post-training block. In the pre- and post-training blocks, participants were instructed to evaluate arguments according to the instructional cues that appeared below the conclusion of conditional statements.

The logic training materials, whilst similar to Experiment 1, were modified so that participants received training for all of the conditional argument structures that were presented in both the first and last block. Thus, we only trained participants with MP and AC arguments. Moreover, unlike in Experiment 1, we also included (pseudo-) invalid arguments, and thus, participants were taught the underlying structures of valid MP (if p then q; p; therefore q), invalid MP (if p then q; p; therefore ¬q), pseudo-valid AC (if p then q; q; therefore p), and pseudo-invalid AC (if p then q; q; therefore ¬p) statements (see Table 1). At the beginning of the training block, participants were asked to do their best to reach 90% accuracy, otherwise, they would be asked to go through the training and the test again. After the training, participants completed the training test, in which they were asked to evaluate the logical validity of 12 abstract conditionals including 6 MP/MP* and 6 AC/AC* problems. Next, participants were presented with the summary of their test performance as in Experiment 1. If the participant’s accuracy was above 90%, she was allowed to proceed to the post-training block. Otherwise, she was informed that her accuracy was below 90% and she has two more chances to reach the desired level. We repeated the same training procedure up to three times and those who failed to reach 90% accuracy even after three attempts were allowed to proceed to the post-training block and finish the experiment. Finally, before proceeding to the post-training block, participants were told that whilst some of the arguments in the training block contained the word “not,” the negations in the following task will be conveyed by using contrasting words.

### Results

#### Logic training test performance

**Fig. 4:**
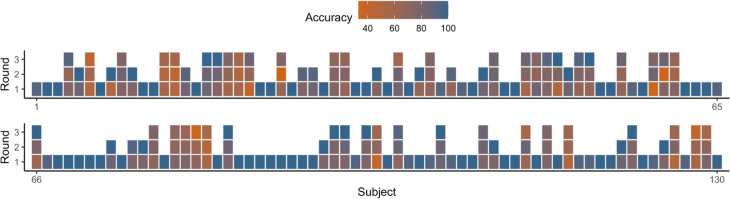
An overview of participants’ accuracy in each round of the logic training test

The new training procedures of Experiment 2 were successful in encouraging participants to engage with the materials, as their accuracy in the training check test reached 90% (*SD* = 16.3), which is notably higher than the training check accuracy of 67% in Experiment 1. The accuracy of MP and AC arguments was 93.1% and 86.8%, respectively. Whilst only 22% of participants (47 out of 202) reached 90% accuracy in Experiment 1, 75% of participants in Experiment 2 had an accuracy above 90%. After three rounds of the training test, 98 participants reached 90% accuracy, with 65 participants in round one, 18 participants in round two, and 15 participants in round three passing the 90% cutoff. The remaining 32 participants failed to reach the target level. Figure 4 summarizes the performance of each participant (*x*-axis) across each training round (*y*-axis). These results indicated that the measures we took to increase participants’ engagement with the training materials were successful.

In analyzing the data of Experiment 2, we included all participants regardless of their accuracy in the training check test. However, a secondary analysis was performed based on excluding participants who failed to reach 90% training test accuracy (*N* = 32). The pattern of the results remained highly similar, in terms of both the magnitude and direction of the focal effects, after excluding those participants (see Tables S1 and S2, and Figs. S1 and S2 in the Supplementary Materials for comparisons of both sets of results).

#### Logic judgments

As in Experiment 1, we first analyze the pre- and post-training trials under logic instructions. The results revealed main effects of (pseudo-) validity (M_post_ = 3.78, HDI [3.21, 4.42]), belief (M_post_ = 1.12, HDI [0.73, 1.53]), argument type (M_post_ = 0.79, HDI [0.47, 1.12]), and block (M_post_ = 0.82, HDI [0.49, 1.20]). Participants showed higher endorsement rates for (pseudo-) valid than (pseudo-) invalid (M_diff_ = 0.94, HDI [0.90, 0.97]), for believable than unbelievable (M_diff_ = 0.43, HDI [0.29, 0.58]), and for logical than pseudo-logical arguments (M_diff_ = 0.31, HDI [0.18, 0.45]). Participants also endorsed more arguments in the pre-training block than the post-training block (M_diff_ = 0.33, HDI [0.20, 0.46]).

**Fig. 5:**
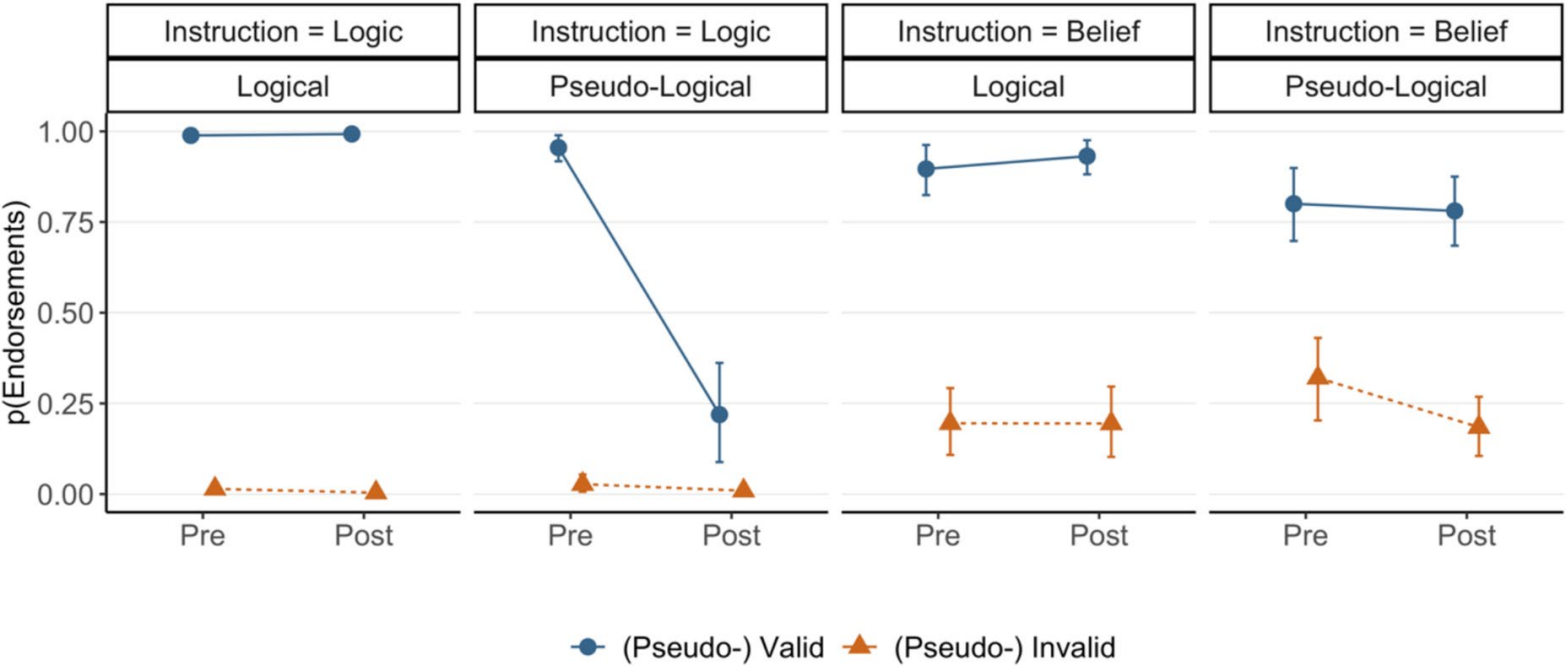
Estimated endorsement ratings of (pseudo) valid and (pseudo-) invalid logical and pseudo-logical arguments under belief and logic instructions across training blocks of Experiment 2. Error bars represent 95% highest density intervals

**Fig. 6:**
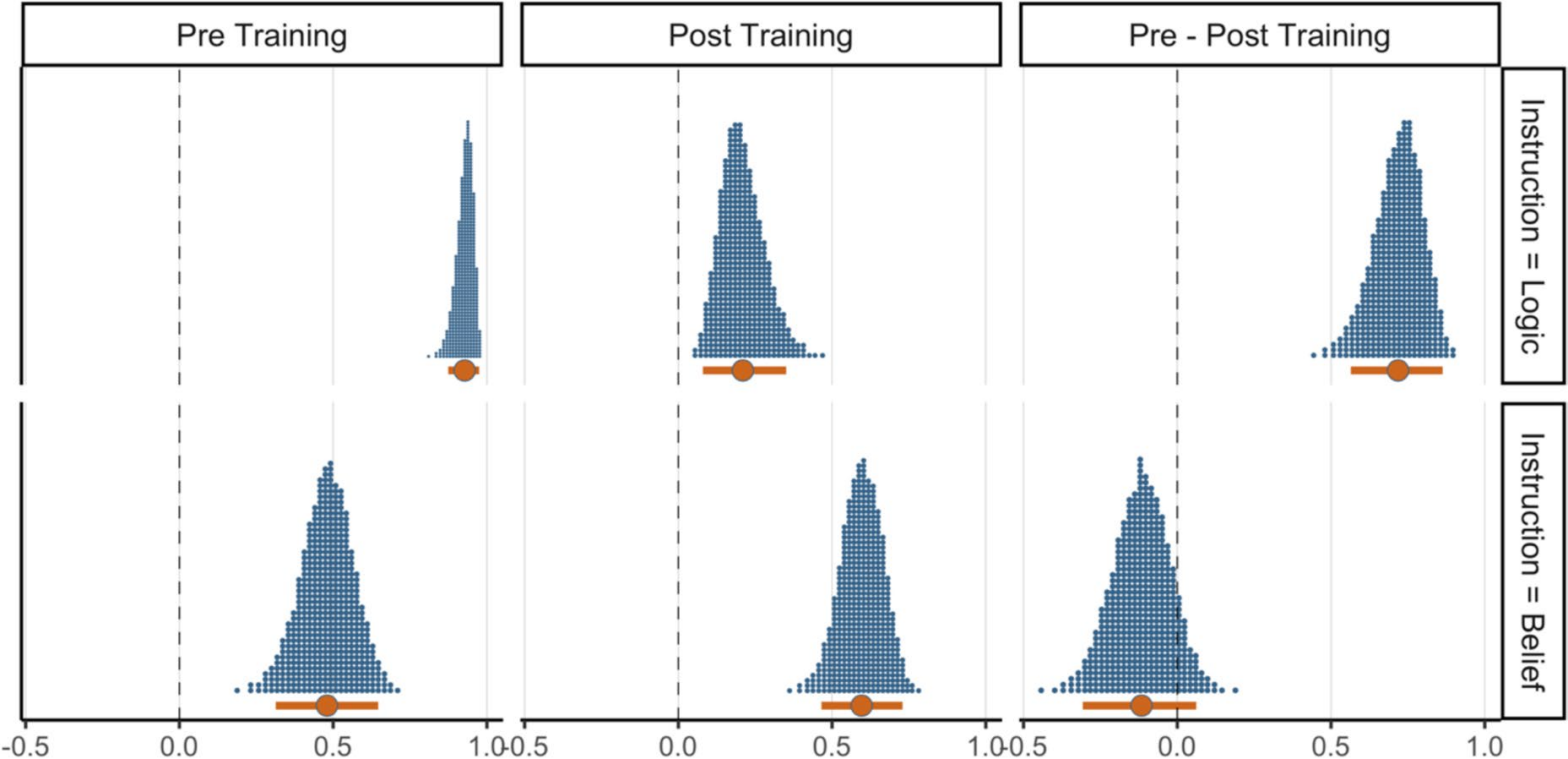
The distributions of validity effects for pseudo-logical arguments under logic and belief instructions of Experiment 2 in each training block along with their contrast. The central circles represent posterior means of the effects and error bars represent 95% HDIs

The results revealed a (pseudo-) validity by argument type interaction (M_post_ = 1.19, HDI [0.83, 1.59]) which indicates a larger (pseudo-) validity effect on logical (M_diff_ = 0.98, HDI [0.97, 0.99]) than pseudo-logical arguments (M_diff_ = 0.70, HDI [0.57, 0.82]). As Fig. 5 suggests, the smaller (pseudo-) validity effect on pseudo-logical arguments is primarily due to the effect of training, as participants showed a substantial decrease in endorsing pseudo-valid arguments in the post-training block. This observation was supported by a credible three-way interaction of (pseudo-) validity, argument type, and block (M_post_ = 0.64, HDI [0.32, 0.99]). In the pre-training block, the (pseudo-) validity effect was similarly large on logical (M_diff_ = 0.97, HDI [0.95, 0.99]) and pseudo-logical (M_diff_ = 0.93, HDI [0.87, 0.98]) arguments. However, after the training, participants rejected pseudo-valid arguments more frequently which resulted in a smaller (pseudo-) validity effect on pseudo-logical (M_diff_ = 0.21, HDI [0.08, 0.35]) than logical arguments (M_diff_ = 0.99, HDI [0.98, 0.99]). Figure 6 highlights the pseudo-validity effects on pseudo-logical arguments before and after training. For logic instructions, this effect was credibly reduced from pre-training (M_diff_ = 0.93, HDI [0.87, 0.98]) to post-training (M_diff_ = 0.21, HDI [0.08, 0.35]), as the HDI of the difference distribution excluded zero as a credible value (M_dod_ = 0.72, HDI [0.57, 0.86]). These findings suggest that a short training session in deductive logic can be highly effective in improving reasoning accuracy for logic judgments. Finally, besides an argument type by block interaction (M_post_ = 0.59, HDI [0.27, 0.93]), no other effects were credibly different from zero.

#### Belief judgments

We performed a separate Bayesian hierarchical regression on belief judgments during pre- and post-training blocks, with (pseudo-) validity, belief, argument type, and block as fixed effects and a maximal random structure. The outputs of the model revealed main effects of (pseudo-) validity (M_post_ = 1.60, HDI [1.27, 1.96]), belief (M_post_ = 2.71, HDI [2.29, 3.16]), and argument type (M_post_ = 0.20, HDI [0.02, 0.38]). Participants endorsed (pseudo-) valid arguments more than (pseudo-) invalid ones (M_diff_ = 0.65, HDI [0.55, 0.74]), believable arguments more than unbelievable ones (M_diff_ = 0.87, HDI [0.81, 0.92]), and logical arguments more than pseudo-logical ones (M_diff_ = 0.09, HDI [0.01, 0.18]).

As in Experiment 1, (pseudo-) validity interacted with argument type (M_post_ = 0.35, HDI [0.15, 0.56]) indicating a somewhat larger validity effect on logical (M_diff_ = 0.72, HDI [0.62, 0.82]) than pseudo-logical arguments (M_diff_ = 0.55, HDI [0.43, 0.67]). The (pseudo-) validity effect, whilst greater on logical arguments, was credibly different from zero on pseudo-logical arguments. This finding suggests that participants are heavily impacted by matching cues in their belief judgments, as a (pseudo-) validity effect with a large effect size was observed on pseudo-logical problems. However, they also rely on logic to some extent as a larger (pseudo-) validity effect was found on logical than pseudo-logical arguments. We return to this finding in the discussion section.

More importantly, training did not modify this effect, and the three-way interaction of (pseudo-) validity, argument type, and block included zero as a credible value (M_post_ = 0.02, HDI [− 0.17, 0.21]), and hence, was not meaningful. Similarly, comparing two models with and without such an interaction term showed that both models had a largely similar predicting accuracy (elpd loo =  − 2627.58, *SE* = 21.43, and elpd loo =  − 2630.74, *SE* = 21.48, for the models without and with the interaction term, respectively).

As Fig. 5 shows, training resulted in lower endorsement rates of pseudo-invalid arguments but it did not affect pseudo-valid arguments. Moreover, the effect of training on sensitivity to validity for logical arguments was also limited. The bottom row of Fig. 6 depicts the validity effect for pseudo-logical arguments under belief instructions, which indicates a non-credible difference in this effect before and after the training (M_dod_ = − 0.12, HDI [− 0.31, 0.06]). This finding, which contradicts the intuitive logic account, indicates that training in the simplest logic rules does not credibly reduce the impact of matching cues on belief judgments. Finally, we found a (pseudo-) validity by belief interaction (M_post_ = 0.30, HDI [0.07, 0.53]) and a belief by argument type interaction (M_post_ = 0.29, HDI [0.10, 0.50]). No other effects were credibly different from zero.

#### State trace analysis

In this section, as an exploratory analysis, we examine whether distinct processes drive logic and belief judgments, whereby the former might primarily rely on Type 2 processing, which is largely affected by validity (with less consideration of believability and matching cues) and the latter mainly relies on Type 1 processing, which is more sensitive to believability and matching cues (with limited sensitivity to validity). Alternatively, a single process—for example, the subjective evaluation of argument strength—could drive both judgments.

**Fig. 7:**
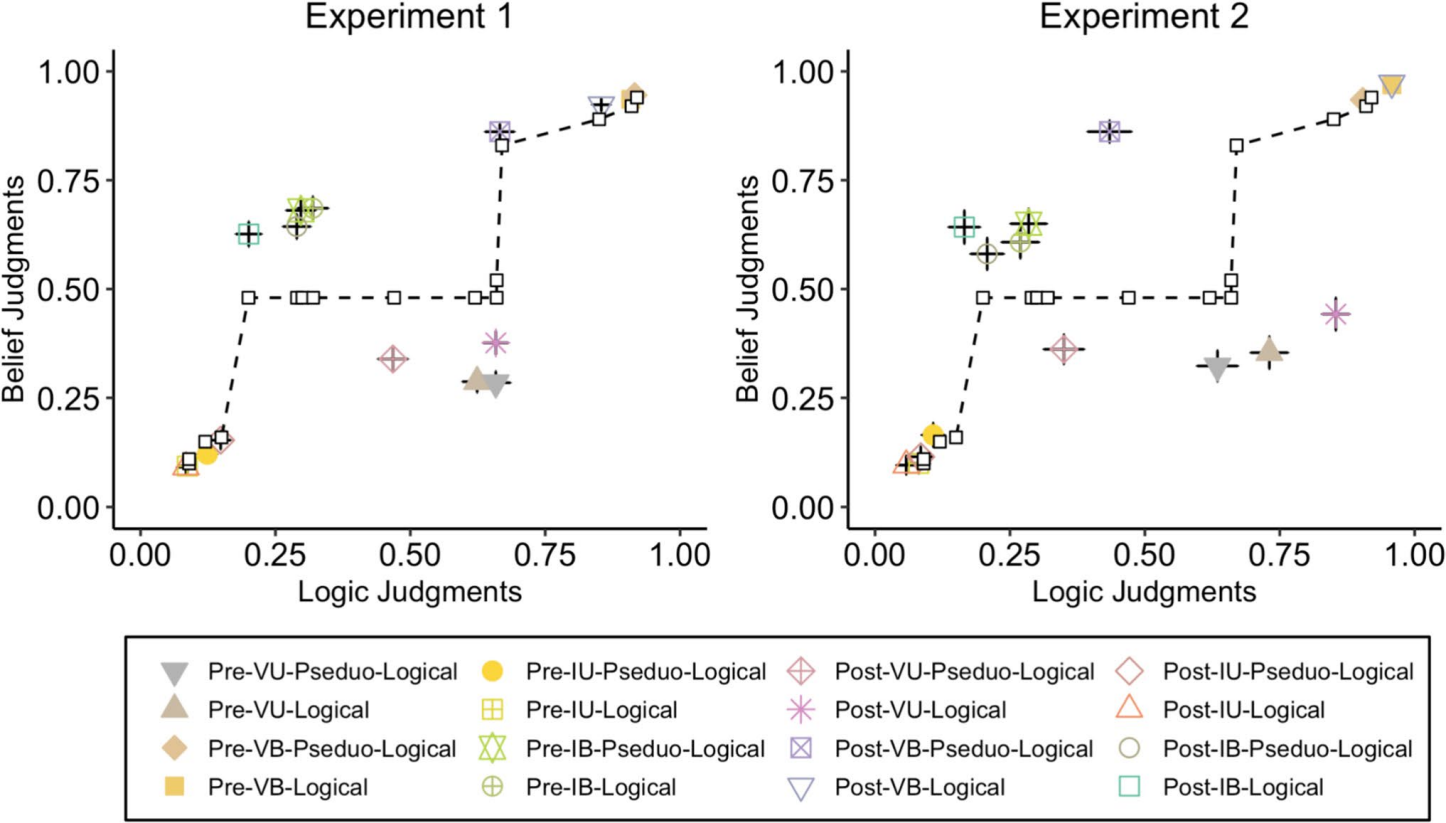
State-trace plots of Experiment 1 (left panel) and 2 (right panel) with logic and belief judgments as dependent variables (*x*- and *y*-axes), and training block, argument type, and argument subtype as independent variables (data points). The dashed line is the best fitting monotonic curve. (Color figure online)

To answer this question, we used state-trace analysis, which is a rigorous method for investigating whether multiple latent variables or processes drive the observed responses (Dunn & Kalish, 2018; Hayes et al., 2018; Newell et al., 2010; Stephens et al., 2019). State-trace analysis makes minimal assumptions about the mapping between the latent (e.g., deliberative and intuitive processes) and dependent (e.g., logic and belief judgments) variables. State-trace analysis assumes merely a monotonic mapping between these variables, with an increase in a latent variable resulting in an increase (or at least, a lack of decrease) in the dependent variable (Stephens et al., 2019). The approach involves examining the relationship between two dependent variables across conditions, which can be visualized using a scatter plot (see Fig. 7). Critically, if only one latent variable drives the dependent variables, the dependent variables must be monotonically related; thus, reliable observations of a nonmonotonic relationship would reject this account in favor of multiple latent variables. Using this analysis approach, Stephens et al. (2019) showed that many findings that were previously assumed to support two qualitatively distinct reasoning processes (namely, intuitive and deliberative processes) can be explained by a single latent variable. We sought to test whether logic and belief judgments might similarly be consistent with a single latent variable, or support multiple processes.

We conducted a separate state-trace analysis for the data of Experiment 1 and 2, with endorsement rates for belief and logic judgments as dependent variables and block (pre- and post-training), argument type (logical and pseudo-logical), and argument subtype (valid-believable, invalid-believable, valid-unbelievable, and invalid-unbelievable) as independent variables that form the conditions. As in the main analyses, we labelled pseudo-logical arguments as valid or invalid, despite the fact that these argument types were all logically invalid. We are thus focusing on the use of a matching cue for logic and belief judgements. Figure 7 depicts state-trace plots of the two experiments, with the dependent variables on the axes and independent variables across the data points in the plot. A visual inspection of the plot suggests a nonmonotonic relationship between logic and belief judgments, as several data points deviate from the best-fitting monotonic curve. This observation was supported by a conjoint monotonic regression analysis (Dunn & Kalish, 2018) which tests the fit of a single latent variable model. The results showed that such a model does not provide a good fit to the observed data (*p* values < 0.001), and hence, distinct cognitive processes may underlie logic and belief judgments.^1^ Therefore, it is possible that although logic instructions encourage an assessment of logical structure and minimize the effect of the matching heuristic (and considerations of believability), belief instructions foster more reliance on believability and element matching, even after extensive training in logic.

### Discussion

The main goal of Experiment 2 was to examine whether a strengthened training intervention would reduce the (pseudo-) validity effect on both belief and logic judgments, which would be evidence against the matching heuristic account that was supported in Experiment 1. Both the results of the training test and the logic judgments in the post-training block revealed that our training intervention was successful in improving reasoning under more deliberative task conditions that instruct participants to assess logic. After training, participants rejected pseudo-logical arguments under logic instructions much more often, such that the size of the (pseudo-) validity effect was significantly reduced. Whilst the logic training was highly effective in boosting logic judgments, it did not suppress the (pseudo-) validity effect for belief judgments; a finding that is consistent with the matching heuristic hypothesis. This finding is in line with the results of Experiment 1, which also found that training selectively reduced the endorsement rates of pseudo-valid arguments for logic judgments but had a limited effect on the corresponding belief judgments.

In contrast to Experiment 1, Experiment 2 also revealed that for belief judgments, participants showed a somewhat larger (pseudo-) validity effect for logical than pseudo-logical arguments. This effect may arise because proposition-matching is the principal problem characteristic that affects belief judgments for pseudo-logical arguments, but both matching and logical cues affect belief judgments for logical arguments—although matching seems to drive most of the effect. Thus, the logic-belief effect for logical arguments may not always entirely arise from a matching heuristic, where matching and logic cues are aligned. In contrast, for pseudo-logical arguments, there is no alignment between logic and matching cues, as all arguments are logically invalid but matching cues vary. Thus, the larger validity effect for belief judgments about logical arguments may be the result of an interplay between logic and matching cues, although logic cues may have only a small influence.

## General discussion

Previous studies have revealed that when participants are instructed to evaluate arguments based on the believability of their conclusions, the belief judgments are impacted by the logical validity of the arguments (Handley et al., 2011; Howarth et al., 2018; Thompson et al., 2018; Trippas et al., 2017). Such a logic-belief effect is considered evidence of intuitive sensitivity to logic and has contributed to revisions of dual-process theories of reasoning, which had traditionally assumed that intuitive Type 1 processing does not have access to rules of logic or probability (Morewedge & Kahneman, 2010; Sloman, 1996). However, contrary to the intuitive logic account, it has been shown that a similar pseudo-logic effect is observed for pseudo-logical arguments which are logically invalid but syntactically matched to equivalent valid or invalid logical arguments (Ghasemi et al., 2022a). Thus, it has been suggested that the logic-belief effect might primarily arise through the operation of a matching heuristic rather than the evaluation of underlying logical structures.

The first goal of the current study was to replicate these findings, confirming previous evidence for matching heuristic hypothesis over the intuitive logic hypothesis. More importantly, the second goal was to investigate whether training in logic selectively enhances the impact of validity for logic judgments—in particular, reducing the impact of pseudo-validity—or whether training also reduces the pseudo-validity effect for belief judgments. Previous research has found that a short training session can be successful at increasing the rate of normatively correct responses, including for either logic judgments or other reasoning judgments made in conditions that foster intuitive thinking (Boissin et al., 2021; Purcell et al., 2020). If the evaluation of logical rules drives the logic-belief effect, then training participants in conditional rules should minimize the pseudo-validity effect under belief instructions, for pseudo-logical arguments. Alternatively, if a matching heuristic underlies the logic-belief effect, training should have little impact on belief judgments.

To test these hypotheses, across two experiments we presented participants with a series of conditional arguments before and after a logic training block and instructed them to evaluate each argument either based on its logical validity or its believability. Half of the arguments were logical arguments with valid and invalid forms. The remaining half were pseudo-logical arguments that were all invalid but included both pseudo-valid and pseudo-invalid forms.

The results of Experiment 1 showed that whilst participants produced a similar pattern of responding to logical and pseudo-logical arguments for both belief and logic judgments before training, they showed higher accuracy in their logic judgments after the training by rejecting pseudo-valid arguments more often. Crucially, training had minimal impact on belief judgments and participants continued to show a strong pseudo-validity effect on pseudo-logical arguments that were all invalid. To boost the training intervention, Experiment 2 focused on simpler arguments, trained participants on all conditional structures used in the experiment and implemented different procedures to enhance participants’ engagement in the training.

The results suggested that the boosted training was more effective and partially replicated the findings of Experiment 1. Whilst participants were impacted by both logical and pseudo-logical structures before training, after training, the effect of the latter was minimized in logic judgments but not belief judgments; training resulted in reduced endorsements of pseudo-logical arguments only under logic instructions. However, unlike in Experiment 1, for belief judgments before training, the effect of validity for logical arguments was somewhat larger than the effect of pseudo-validity for pseudo-logical arguments. Nevertheless, these (pseudo-) validity effects on belief judgments were unaffected by training. These results largely support the matching heuristic account, according to which, the simple consideration of element matching rather than logic assessment primarily underlies previous evidence for intuitive logic.

One might argue that training failed to do so simply because our training intervention was not adequate to automatize the newly learned logic structures and hence, build mindware. It has been shown that such mindware predicts responses consistent with the use of intuitive logic over and above reasoning ability and intelligence (Šrol & De Neys, 2020). However, as noted at the outset, the logic training was not intended to build new mindware, but primarily to clarify the structure of simple logical structures and to activate previously learned knowledge of conditional rules. Such “If p then q” knowledge is learned through years of formal education (e.g., “If a number is divisible by 2, then it is an even number”), daily experimentation (e.g., “If you boil the water, then it evaporates”), and everyday conversations (“If we win the final match, then we celebrate”). Indeed, our training procedures, especially in Experiment 2, were highly effective for debiasing logic judgments. Thus, the lack of a clear training effect on belief judgments in the current study more likely results from a highly dominant matching heuristic, rather than inadequate training. However, future research could test more extended training procedures.

### Matching heuristic account

The findings of our study demonstrate that a matching heuristic could be a key mechanism behind apparent logic-belief effects. According to this account, under task conditions thought to foster more intuitive processing, such as the belief instructions used in our experiments, participants are often influenced by matching cues rather than logical structure. For example, the operation of this matching heuristic leads to greater rates of endorsement for both MP (If p then q; p; therefore q) and AC (If p then q; q; therefore p) arguments (even though AC is invalid) than both MP* (If p then q; p; ¬q) and AC* (If p then q; q; ¬p) arguments, even when participants are simply asked to evaluate conclusion believability rather than logical validity. The premises of MP and AC arguments are syntactically matched with their conclusions, as p and q propositions in the premises match the p and q propositions in the conclusion. Such matching is absent for MP* and AC* arguments; there is no match between the p and q propositions in the premises and the negated proposition in the conclusion.

Upon the presentation of a reasoning problem, multiple cues such as conclusion believability, proposition matching, or logical validity are typically available to influence reasoning judgments. Under the matching heuristic account, in situations where more intuitive processing may be fostered, as with belief instructions, proposition matching plays a large role in driving responses. We would argue that matching dominates as a more rapid, intuitive influence because it likely preempts any logical processing, probably through an associative process that draws upon automatic pattern recognition capability (Rao & Ballard, 1999) or well-practiced language comprehension (Krzyżanowska et al., 2017; Stephens et al., 2020; Trippas et al., 2016). This dominance of matching cues over logic cues was supported by an additional analysis comparing arguments with aligned and misaligned logic and matching cues. Under belief-based instructions, participants endorsed both valid-match and invalid-match arguments at similar rates, and training had little effect in differentiating these conditions, suggesting that individuals relied primarily on matching cues, endorsing matched arguments regardless of logical validity. Under logic-based instructions, a similar pattern emerged initially, but after training, participants were significantly less likely to endorse invalid-match arguments, indicating that training reduced reliance on the matching heuristic. Furthermore, linear regression models predicting endorsement rates under belief-based instructions showed that belief had the strongest effect, followed by matching, while logic had the weakest influence. The effect of logic was nonsignificant in Experiment 1 and only marginally significant in Experiment 2 before training, with minimal improvements after training (see Figs. S11 and S12 in the Supplementary Materials for further details). A key limitation of these analyses is that the partial alignment of matching and logic cues makes it difficult to isolate their individual contributions.

However, individual differences may exist in how cues are weighted. While matching cues exert the greatest influence in this task, a subset of participants may give greater weight to logical cues, especially after training facilitates the activation of logical structures. Figures S9 and S10 in the Supplementary Materials illustrate the distribution of the difference in endorsement rates between (pseudo-)valid and (pseudo-)invalid arguments for logical and pseudo-logical conditions. These figures suggest that a small subset of participants exhibited a stronger logic-belief effect for logical than pseudo-logical arguments after training, supporting the presence of individual differences in cue weighting. Future studies could explore these differences more robustly by using arguments where logic and matching cues lead to conflicting responses.

Another important question raised by our results is whether logic judgments are also impacted by a similar matching heuristic—at least prior to any logic training—in the same way as belief judgments are? Indeed, in line with previous research on deductive reasoning (Ghasemi et al., 2022a, 2023; Meyer-Grant et al., 2022), our results showed that in the pre-training block, there was a sizable (pseudo-) validity effect on pseudo-logical arguments under logic instructions. This effect was of a comparable size to the validity effect for logical arguments. The role that matching heuristics play in reasoning has been the focus of research for more than 50 years, particularly in the context of the “matching bias” phenomenon in Wason’s selection task (Wason, 1968). In this task, participants are given a rule (e.g., “If there is an A on one side of the card then there is a 3 on the other side”) and four cards that have a number and a letter on each side (e.g., “A” or “L” on one side; “3” or “7” on the other). Multiple studies (e.g., Evans & Lynch, 1973; Oaksford & Chater, 1994; Thompson et al., 2013) have shown that when people are asked to select those card(s) that need to be turned over in order to determine whether the rule is true or false, people tend to pick the cards that were included in the rule statement (i.e., “A” and “3”) instead of the logically correct cards (i.e., “A” and “7”). Interestingly, the tendency to select matching cards persists even when the conditional rule contains negated components, leading to correct selections on certain rules (e.g., “If there is an A on one side of the card then there is *not* a 3 on the other side”), where the selection of the matching “A” and “3” cards serves to falsify the rule. The observation that matching bias can lead to accurate responding on some problems but inaccurate responding on others mirrors our current observations, whereby before training, participants fail to discriminate between some valid and invalid arguments but not others in their responding under logical instructions. The differential impact of matching bias on accuracy has also been observed for syllogistic reasoning tasks, where reasoners tend to prefer conclusions in which the form of the quantifier matches one of the quantifiers in the problem premises (Chater & Oaksford, 1999; Wetherick & Gilhooly, 1995). This can lead to accurate responding for problems in which a matching conclusion aligns with a logical one but poor performance where they are misaligned.

In our study, it is quite likely that before training, logic judgments are influenced by a matching heuristic in the same way that such judgments are affected by the believability of the argument’s conclusion (in the case of belief bias). The reduction in the endorsement of pseudo-logical arguments after training might suggest that training can be successful in minimising the influence of a matching heuristic on logic judgments, shifting participants to engage in a more deliberative logical reasoning strategy. This claim is supported by the results of the state-trace analyses, which confirm that more than one latent psychological variable drives logic and belief judgments. It is possible that logic judgments depend more strongly on deliberative assessment of logical validity, while belief judgments depend more strongly on intuitive assessments of proposition-matching and conclusion believability.

### Other possible alternative accounts

A core finding of the current study is that, under belief instructions, the endorsement of the conclusions to pseudo-logical arguments mirrors that of logical arguments. Thus far, we have claimed that this finding can be explained by a matching heuristic account, according to which participants’ judgments are influenced by the presence of a match between the premise and conclusion propositions. However, one alternative explanation of the logic-belief effect could be based on probabilistic accounts of reasoning. According to these accounts, reasoners draw inferences based upon the probabilistic characteristics of an argument, where strong arguments are ones in which the premises guarantee the conclusion with a high degree of probability (Oaksford et al., 2000). Perhaps participants endorse the conclusions to (pseudo-) valid arguments more than the conclusions to (pseudo-) invalid arguments because the former are inductively stronger than the latter. As already explained, all the pseudo-logical arguments were logically invalid, but half of them (pseudo-valid) were possible-strong arguments that are often endorsed by individuals, and the other half (pseudo-invalid) were possible-weak arguments that are regularly rejected (Evans et al., 2010). Therefore, it is possible that participants apply a probabilistic approach in evaluating the strength of association between conditional propositions instead of a content-independent logic assessment or a syntactic-matching heuristic—this possibility could be explored in future. However, other candidate accounts may be more important to consider, such as the dual-source model that has been shown to outperform probabilistic models (Singmann et al., 2016).

According to the dual-strategy (Markovits et al., 2020) and dual-source (Singmann et al., 2016) models, participants either use a counterexample-based strategy or a content-dependent statistical strategy in processing conditional arguments. It is quite possible that many individuals primarily rely on their prior knowledge, and hence a statistical approach, in their belief and logic judgments. Moreover, whilst logic training may prompt individuals to switch to a counterexample-based strategy for logic judgments, the statistics-based approach might remain the primary influence on responding under belief instructions. Such a dissociation between two sources of information may also be consistent with the results of the state-trace analyses that suggest more than one type of processing underlies judgments under logic and belief instructions. Future studies could usefully shed further light on the underlying reasoning mechanisms by pitting the predictions of the matching heuristic and dual-source models against each other.

Another possible account of belief and logic judgments could be a single-process model, which posits that both judgments are driven by a common subjective assessment of argument strength, rather than distinct Type 1 and 2 processing. Previous work has shown that such models, drawing on the signal detection framework, can account for a wide range of data—including training effects—based on logic judgments versus inductive judgments, where the latter involves participants judging the plausibility of an argument’s conclusion based on the premises (Stephens et al., 2018, 2020). Single-process signal detection models have not yet been tested against logic versus belief judgments, and such judgments might offer a stronger test of the models. Importantly, our state-trace analysis results suggest that a successful single-process model might require more than one parameter (noting that our conditions were based on (pseudo-) validity rather than only validity). For example, one candidate model that could be tested in future work is the “independent- 1D” model advanced by Stephens et al. (2018), which includes a common discriminability parameter but independent decision criteria parameters for each judgment type.

Finally, it is possible that reasoners had a biconditional interpretation of conditionals, which entails both MP and AC arguments as truly, yet biconditionally, valid arguments, as “p implies q” and “q implies p.” This explanation challenges the matching heuristic account; reasoners still rely on logical validity in their intuitive reasoning, but the logic is a biconditional rather than a conditional type. Previous studies, however, ruled out this possibility by showing different response patterns for MP and AC arguments, indicating that a biconditional reading of conditional propositions is less frequent than assumed. For example, it has been shown that the endorsement ratings of AC decrease where there is a strict emphasis on logical necessity (Evans et al., 2010), or under time constraints (Newman et al., 2017). Even without such interventions, studies have found that people tend to endorse MP arguments more than AC ones and reject MP* more than AC* ones, indicating limited evidence of biconditional evaluation of conditional arguments (Ghasemi et al., 2022a). In order to test the biconditional hypothesis using our own data, we compared the endorsement ratings of “MP” and “AC” arguments under logic instructions and found a more marked validity effect on MP arguments compared with AC arguments, demonstrating a higher endorsement of MP than AC arguments, and higher rejection of MP* than AC* arguments (see the Supplementary Materials for more detailed results). This finding is consistent with evidence in the literature against a biconditional interpretation of conditionals.

### Implications for intuitive logic accounts

Previous research seemed to have shown that participants are intuitively sensitive to logic, as the validity of an argument’s conclusion interferes with belief, liking, and brightness judgments (Ghasemi et al., 2021; Morsanyi & Handley, 2012; Thompson et al., 2018; Trippas et al., 2016, 2017). These effects have been presumed to occur without conscious awareness through the automatic activation of simple logical rules or principles and has consequently been named “intuitive logic” or “logical intuition” by recent dual-process theorists (Handley & Trippas, 2015; Thompson & Newman, 2017; but see Hayes et al., 2020, 2022). Related effects have been studied extensively across different research paradigms (e.g., instructional manipulation, conflict detection, and a two-response paradigm), multiple tasks (e.g., liking, brightness, logic, or belief judgments of arguments, and ratio-bias tasks), normative rules (e.g., logic, probability, math), and reasoning problems (e.g., syllogisms, base-rate, CRT problems).

Although these studies appear to have established a firm foundation of support for the role of intuitive logic in reasoning, there remains limited evidence that critical effects such as the logic-belief effect genuinely reflect intuitive sensitivity to logical or other normative rules. Indeed, recent research suggests that more superficial problem features, such as propositional matching, rather than logic underlie the logic-belief effect (Ghasemi et al., 2023, 2022a). The current study replicated and extended the key findings of Ghasemi et al., (2022a) study, showing that matching could play a large role in belief judgments, while considerations of logic have minimal influence. Thus, the logic-belief effect, which once was assumed to mainly arise from intuitive sensitivity to logic, is found to primarily be a product of sensitivity to more superficial characteristics of an argument such as matching of its elements. We have acknowledged that participants may not solely rely on matching and it is still possible that some participants process the logical validity of arguments when making belief judgments. However, sensitivity to matching cues seems to be the primary driver of the logic-belief effect for untrained reasoning and such cue use is affected little by logic training, suggesting that genuine logical intuition is rare at best.

These findings call for further research to understand the nature of intuitive reasoning more precisely. Claims about logical intuitions are not limited to research on deductive reasoning but have been made for other reasoning problems too, such as the base-rate, ratio-bias, and CRT tasks. Whilst the structure of these judgment tasks means that a matching heuristic is an unlikely underlying mechanism of apparent intuitive influences in these cases, it is nevertheless quite possible that reasoners are sensitive to other relatively superficial problem features, and it is these rather than an appreciation of normative rules that influence judgments. For the base-rate task, for example, a heuristic that simply compares the relative numerical base-rate values and selects the option consistent with the largest number would be sufficient to deliver a normative response. This would not require any deep appreciation of normative rules such as Bayes theorem, because the solution does not require integration of prior probabilities with diagnostic evidence.

An important task for future research will be to extend the current work to determine the extent to which apparent logical intuitions rely upon simple heuristic strategies across a broader range of tasks. It is also worth noting that individual reasoners might vary in the extent to which they draw upon heuristic or logical strategies, and it is possible that a subset of reasoners are able to intuit based upon logico-mathematical principles, whereas other reasoners are influenced more significantly by simple heuristics (Thompson & Markovits, 2021). Therefore, future research needs to further investigate individual differences and task-dependent factors that influence the extent to which reasoners weight different types of cues. Top-down characteristics such as cognitive ability, thinking style, mindware, and conflict detection efficiency (Šrol & De Neys, 2020) are possible candidates that may cause individual differences in a cue-weighting process. An interesting research query is to examine if such individual differences are quantitative (i.e., all participants weigh matching cues more than logic cues) or qualitative (i.e., a subgroup of participants weigh logic cues more than matching ones). This type of research question could be readily addressed through designing tasks in which a simple heuristic or a logical strategy will result in different behavioural responses. A Bayesian model comparison approach is needed to take measurement errors into account and to pit different constrained and unconstrained models against each other (Haaf & Rouder, 2019). Besides these top-down cognitive factors, several possible task features might affect cue weighting, such as problem complexity, the coherency of elements, the nature of the instructions, or even physical characteristics such as the brightness of statements (Hayes et al., 2022).

Future findings, along with the current results, can inform intuitive logic accounts, and more broadly, dual-process theories of human reasoning. If logic can only explain a small proportion of apparent intuitive logic effects, then theoretical explanations need to take into account the matching heuristic or other simple strategies as primary mechanisms.

### Potential limitations

One possible concern could be that the smaller pre-training effect of (pseudo-) validity under belief instructions than under logic instructions has reduced the scope to observe the effect of training. Under belief instructions, participants were asked to evaluate the believability of arguments’ conclusions and hence a smaller impact of (pseudo-) validity is expected compared with logic instructions, which makes the issue difficult to avoid. Nevertheless, in both experiments, participants showed a substantial (pseudo-) validity effect in their belief judgments before training, so there was much scope for training to have an impact, especially given the sizeable impact of training on both logic judgments and the training test.

Moreover, since we used a within-subject design with logic and belief instructional cues that are in conflict in half of the trials, one may argue that the interference of syntactic structures, whether logic or matching, on belief judgments is a by-product of a task-switch strategy. According to this hypothesis, a trial-by-trial switching between logic and belief instructions, rather than the conflict between matching/logic and belief status of an argument, may result in the interference effect. Such a hypothesis has been discussed and challenged in previous studies. By using a between-subject design, studies have found the same pattern of results as experiments with a within-subject design (e.g., Handley et al., 2011; Pennycook et al., 2014; Howarth et al., 2016). To further rule out the task-switch hypothesis using our own data, we performed an analysis by labelling our trials as “switch” (trials in which participants were cued with a logic/belief instruction different than the previous trial) and “repeat” (trials in which participants were cued with the same instruction as the previous trial). Our analysis showed that this factor did not interact with (pseudo-) validity and/or instruction (see the Supplementary Materials for a more detailed discussion).

## Conclusion

Recent theories of human reasoning have argued that individuals can be intuitively sensitive to logical rules as, when they are simply instructed to evaluate the believability of an argument’s content, the logical validity of the argument interferes with their belief judgments. Such an intuitive grasp of logic rules has been challenged by findings showing that the interference could be mainly caused by the matching of argument propositions rather than their logical structures because syntactically matched pseudo-logical arguments create a pseudo-validity effect of a similar magnitude. Moreover, training individuals in deductive reasoning, whilst successful in improving logic judgments, does not reduce the reliance on element matching for belief judgments. Altogether, these findings suggest that apparent logical intuitions are often driven by a superficial matching heuristic rather than an assessment of logic itself.

## Supplementary Information

Below is the link to the electronic supplementary material.

Supplementary file1 (DOCX 5588 KB)

## Data Availability

All materials and data (Ghasemi et al., 2022b) have been made publicly available at the Open Science Framework website and can be accessed at (https://osf.io/r382w/).

## Footnotes

1 It should be noted that we tested a simple single-process model with only one latent variable for both logic and belief judgments. However, as Stephens et al. (2018) demonstrated, more complex single-process models are possible. For example, a single-process model based on the signal detection framework could have a discriminability parameter, plus a distinct decision criterion for each dependent variable, which might provide a good fit for the current experimental data.

## Code availability

The code used for analysis in this study is available at the Open Science Framework (https://osf.io/r382w/).

## Declarations

### Open science statement

All materials, analysis codes, and data (Ghasemi et al., 2022b) have been made publicly available at the Open Science Framework website and can be accessed at (https://osf.io/r382w/).
