# Supplementary material for: Logical intuitions or matching heuristics? Examining the effect of deduction training on belief-based reasoning judgments
Source: Mem Cognit. 2025 Apr 11;53(7):2279–300. doi: 10.3758/s13421-025-01710-3 (PMC12589337; doi:10.3758/s13421-025-01710-3)
Supplement: Supplementary file 1 — Supplementary file1 (DOCX 5588 KB) [file 13421_2025_1710_MOESM1_ESM.docx]

Logical intuitions or matching heuristic? Examining the effect of deduction training on belief-based reasoning judgments (Supplementary Materials)

Omid Ghasemi, Simon J. Handley, Rachel G. Stephens

Table of Contents

[1 Authors’ Notes 1](#_Toc193093829)

[2 Materials 1](#_Toc193093830)

[3 Instructions 2](#_Toc193093831)

[4 Baysian Analysis 3](#_Toc193093832)

[4.1 Re-analysis of Experiment 2 with Excluding Failed Participants 3](#_Toc193093833)

[4.2 Model Specifications 6](#_Toc193093834)

[4.3 Model Outputs 8](#_Toc193093835)

[4.4 Postrior Predictive Checks 9](#_Toc193093836)

[4.5 Trace Plots 10](#_Toc193093837)

[4.6 Model Comparison 10](#_Toc193093838)

[5 Further Analyses 11](#_Toc193093839)

[5.1 Task-Switch Hypothesis 11](#_Toc193093840)

[5.2 Biconditional Reading of Conditionals 12](#_Toc193093841)

[5.3 Descriptive Results 13](#_Toc193093842)

[5.4 Individual Differences 14](#_Toc193093843)

[5.5 Matching vs. Logic 15](#_Toc193093844)

# 1 Authors’ Notes

All materials, data, and analysis scripts can be retrieved at the Open Science framework (<https://osf.io/r382w/>).

All correspondence related to this project should be addressed to:

**Omid Ghasemi** (University of New South Wales, Sydney, NSW, AUSTRALIA)

Email: [o.ghasemi@unsw.edu.au](mailto:o.ghasemi@unsw.edu.au)

# 2 Materials

- Materials including reasoning problems and the logic training can be retrieved at the Open Science framework (<https://osf.io/t82hu/>).
- We have hosted the logic training blocks of both experiments on GitHub and you can do the training [here](https://omidghasemi21.github.io/Results/logic_training_exp1/index.html) (Experiment 1) and [here](https://omidghasemi21.github.io/Results/logic_training_exp2/index.html) (Experiment 2).

# 3 Instructions

*People usually use two types of reasoning: reasoning on the basis of LOGIC or reasoning on the basis of their BELIEFS. We aim to examine these two types of judgments on three tasks. In each trial of the first task, you will be presented with an argument with two premises (sentences/statements) above a line and, after a short amount of time, a conclusion below the line. You should evaluate each argument on the basis of logical validity or believability according to a cue (LOGIC or BELIEF) which will be presented in red at the bottom of the screen.*

*When the cue is “LOGIC”, you need to assume the two premises are true (even if they do not make sense in real world) and you should judge whether the conclusion necessarily follows from those premises. You can choose your response by clicking on the “Valid” box if you think the argument is logically valid and the “Invalid” box if you think the argument is logically invalid. For example:*

*If it is night time, then the sky is bright
It is night time
The sky is bright*

*According to our knowledge of the world, we know that the sky is black and dark during the night, however, this problem is logically valid because its conclusion necessarily follows from its premises.*

*On the other hand, when the cue is “Belief”, you should evaluate the conclusion based on what you think is true in the real world. By considering the congruency of the conclusion to your general knowledge, you should select the “Believable” box if you think the conclusion is generally believable and select the “Unbelievable” box if you think the conclusion does not make any sense. For example:*

*If a singer has a sore throat, then her singing will be nice
The singer has a sore throat
Her singing will be bad*

*Given the “BELIEF” cue, the conclusion is believable because you know from your knowledge of the world and personal experience that a singer is unlikely to sing well when she has a sore throat.*

*Before starting the main part of the experiment, you can check your understanding of the task and the instructions by running through several practice items. Click Next to begin the practice items.*

# 4 Baysian Analysis

## 4.1 Re-analysis of Experiment 2 with Excluding Failed Participants

In Experiment 2, at the beginning of the training block, participants were informed that they needed to reach 90% accuracy in the training check test to be able to proceed to the next block, and if accuracy was lower than 90%, they would be returned to the beginning of the training block. If the participant’s accuracy was above 90%, she was allowed to proceed to the post-training block. Otherwise, she was informed that her accuracy was below 90% and she has 2 more chances to reach the desired level. We repeated the same training procedure up to 3 times and those who failed to reach 90% accuracy even after 3 attempts were allowed to proceed to the post-training block and finish the experiment.

Whilst only 22% of participants (47 out of 202) reached 90% accuracy in Experiment 1, 75% of participants in Experiment 2 had an accuracy above 90%. After 3 rounds of the training test, 98 participants reached 90% accuracy, with 65 participants in round one, 18 participants in round two, and 15 participants in round three passing the 90% cutoff. The remaining 32 participants failed to reach the target level. In the main results section of Experiment 2, we analysed all participants regardless of their accuracy in the training check test.

In this section, we excluded those 32 participants who failed to reach 90% accuracy even after 3 attempts to see if the results remain the same. To do so, we conducted the same Bayesian hierarchical logistic regression models on the remaining 98 participants. Tables below summarise the outputs of both full and excluded models for logic (Table S1) and belief (Table S2) judgments. As you can see, the results are highly similar, especially for belief judgments.

Table S1: The outputs of full and excluded models for logic judgments in Experiment 2

| **Term** | **Model** | **Estimate** | **SD** | **CI Low** | **CI High** |
| --- | --- | --- | --- | --- | --- |
| (Intercept) | Excluded | -1.418 | 0.314 | -2.085 | -0.842 |
| (Intercept) | Full | -0.887 | 0.207 | -1.311 | -0.497 |
| argument_type1 | Excluded | 1.499 | 0.279 | 0.989 | 2.080 |
| argument_type1 | Full | 0.785 | 0.165 | 0.472 | 1.123 |
| argument_type1:block1 | Excluded | -1.076 | 0.280 | -1.653 | -0.557 |
| argument_type1:block1 | Full | -0.588 | 0.166 | -0.931 | -0.273 |
| belief1 | Excluded | 1.273 | 0.314 | 0.694 | 1.923 |
| belief1 | Full | 1.118 | 0.203 | 0.734 | 1.532 |
| belief1:argument_type1 | Excluded | 0.257 | 0.216 | -0.162 | 0.699 |
| belief1:argument_type1 | Full | 0.110 | 0.140 | -0.152 | 0.402 |
| belief1:argument_type1:block1 | Excluded | 0.074 | 0.206 | -0.336 | 0.484 |
| belief1:argument_type1:block1 | Full | -0.018 | 0.118 | -0.254 | 0.215 |
| belief1:block1 | Excluded | 0.345 | 0.237 | -0.115 | 0.817 |
| belief1:block1 | Full | 0.235 | 0.152 | -0.066 | 0.533 |
| block1 | Excluded | 1.339 | 0.289 | 0.816 | 1.948 |
| block1 | Full | 0.820 | 0.179 | 0.489 | 1.195 |
| pseudo_logic1 | Excluded | 5.205 | 0.551 | 4.216 | 6.377 |
| pseudo_logic1 | Full | 3.779 | 0.312 | 3.209 | 4.425 |
| pseudo_logic1:argument_type1 | Excluded | 1.800 | 0.308 | 1.243 | 2.455 |
| pseudo_logic1:argument_type1 | Full | 1.190 | 0.191 | 0.839 | 1.587 |
| pseudo_logic1:argument_type1:block1 | Excluded | -1.048 | 0.279 | -1.631 | -0.537 |
| pseudo_logic1:argument_type1:block1 | Full | -0.642 | 0.171 | -0.991 | -0.317 |
| pseudo_logic1:belief1 | Excluded | -0.068 | 0.228 | -0.529 | 0.374 |
| pseudo_logic1:belief1 | Full | -0.057 | 0.156 | -0.373 | 0.237 |
| pseudo_logic1:belief1:argument_type1 | Excluded | 0.043 | 0.202 | -0.353 | 0.442 |
| pseudo_logic1:belief1:argument_type1 | Full | 0.017 | 0.113 | -0.203 | 0.245 |
| pseudo_logic1:belief1:argument_type1:block1 | Excluded | -0.211 | 0.204 | -0.616 | 0.183 |
| pseudo_logic1:belief1:argument_type1:block1 | Full | -0.103 | 0.109 | -0.319 | 0.109 |
| pseudo_logic1:belief1:block1 | Excluded | 0.242 | 0.212 | -0.175 | 0.657 |
| pseudo_logic1:belief1:block1 | Full | 0.207 | 0.131 | -0.044 | 0.469 |
| pseudo_logic1:block1 | Excluded | 0.307 | 0.312 | -0.300 | 0.938 |
| pseudo_logic1:block1 | Full | 0.189 | 0.199 | -0.196 | 0.583 |

Table S2: The outputs of full and excluded models for belief judgments in Experiment 2

| **Term** | **Model** | **Estimate** | **SD** | **CI Low** | **CI High** |
| --- | --- | --- | --- | --- | --- |
| (Intercept) | Excluded | 0.382 | 0.176 | 0.047 | 0.735 |
| (Intercept) | Full | -0.887 | 0.207 | -1.311 | -0.497 |
| argument_type1 | Excluded | 0.411 | 0.132 | 0.160 | 0.673 |
| argument_type1 | Full | 0.785 | 0.165 | 0.472 | 1.123 |
| argument_type1:block1 | Excluded | -0.117 | 0.126 | -0.361 | 0.136 |
| argument_type1:block1 | Full | -0.588 | 0.166 | -0.931 | -0.273 |
| belief1 | Excluded | 3.153 | 0.299 | 2.597 | 3.788 |
| belief1 | Full | 1.118 | 0.203 | 0.734 | 1.532 |
| belief1:argument_type1 | Excluded | 0.325 | 0.134 | 0.070 | 0.602 |
| belief1:argument_type1 | Full | 0.110 | 0.140 | -0.152 | 0.402 |
| belief1:argument_type1:block1 | Excluded | -0.060 | 0.132 | -0.318 | 0.201 |
| belief1:argument_type1:block1 | Full | -0.018 | 0.118 | -0.254 | 0.215 |
| belief1:block1 | Excluded | 0.095 | 0.160 | -0.219 | 0.417 |
| belief1:block1 | Full | 0.235 | 0.152 | -0.066 | 0.533 |
| block1 | Excluded | 0.163 | 0.145 | -0.123 | 0.454 |
| block1 | Full | 0.820 | 0.179 | 0.489 | 1.195 |
| pseudo_logic1 | Excluded | 1.856 | 0.242 | 1.397 | 2.338 |
| pseudo_logic1 | Full | 3.779 | 0.312 | 3.209 | 4.425 |
| pseudo_logic1:argument_type1 | Excluded | 0.464 | 0.139 | 0.198 | 0.742 |
| pseudo_logic1:argument_type1 | Full | 1.190 | 0.191 | 0.839 | 1.587 |
| pseudo_logic1:argument_type1:block1 | Excluded | 0.014 | 0.131 | -0.238 | 0.275 |
| pseudo_logic1:argument_type1:block1 | Full | -0.642 | 0.171 | -0.991 | -0.317 |
| pseudo_logic1:belief1 | Excluded | 0.388 | 0.158 | 0.086 | 0.702 |
| pseudo_logic1:belief1 | Full | -0.057 | 0.156 | -0.373 | 0.237 |
| pseudo_logic1:belief1:argument_type1 | Excluded | 0.207 | 0.130 | -0.033 | 0.473 |
| pseudo_logic1:belief1:argument_type1 | Full | 0.017 | 0.113 | -0.203 | 0.245 |
| pseudo_logic1:belief1:argument_type1:block1 | Excluded | 0.061 | 0.120 | -0.168 | 0.298 |
| pseudo_logic1:belief1:argument_type1:block1 | Full | -0.103 | 0.109 | -0.319 | 0.109 |
| pseudo_logic1:belief1:block1 | Excluded | 0.254 | 0.140 | -0.011 | 0.538 |
| pseudo_logic1:belief1:block1 | Full | 0.207 | 0.131 | -0.044 | 0.469 |
| pseudo_logic1:block1 | Excluded | -0.052 | 0.153 | -0.347 | 0.259 |
| pseudo_logic1:block1 | Full | 0.189 | 0.199 | -0.196 | 0.583 |

Figure S1 shows the pattern of the results with (excluded model) and without (full model) excluding participants who failed to reach 90% accuracy in the training check test.


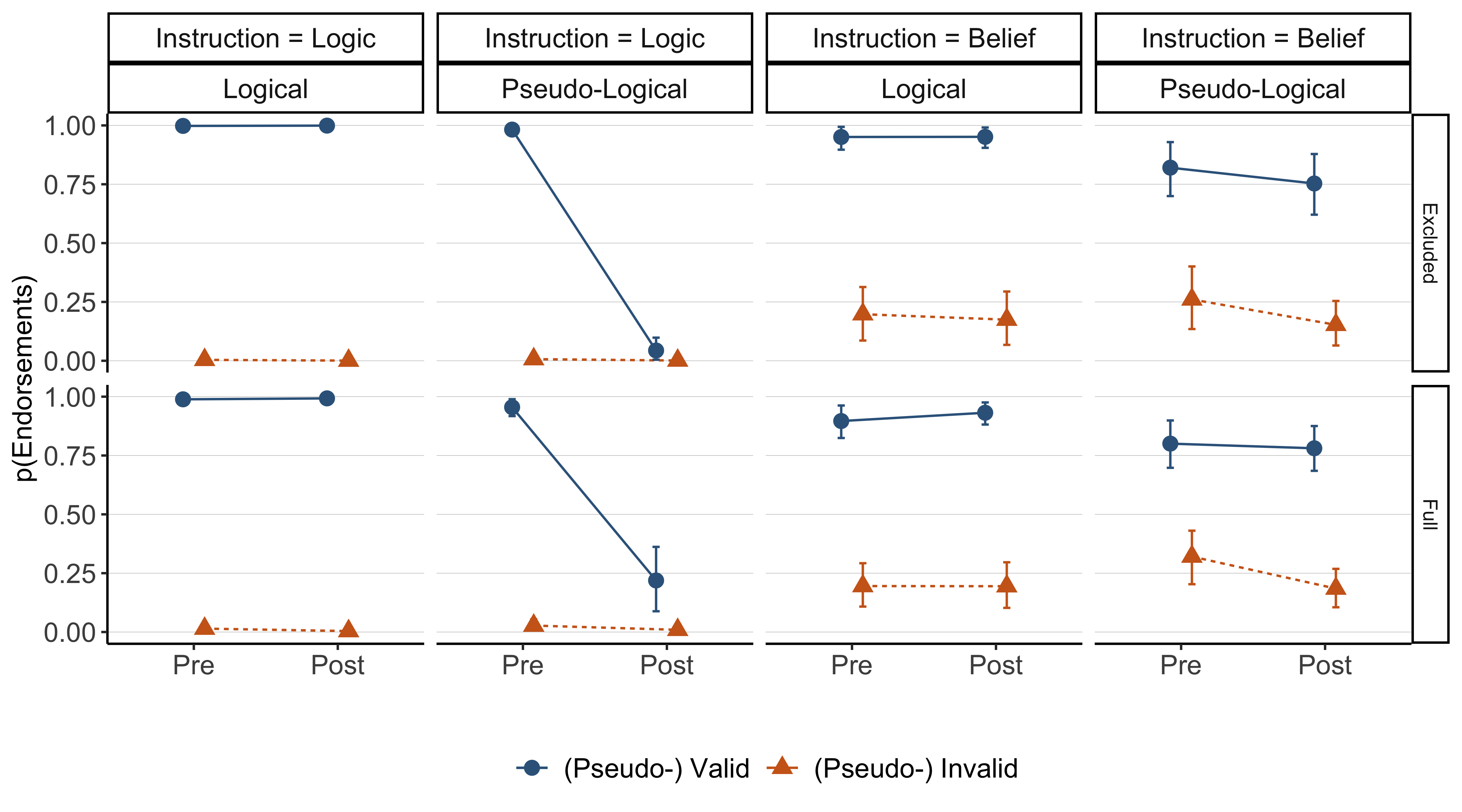


Figure S1: Estimated endorsement ratings of (pseudo) valid and (pseudo-) invalid logical and pseudo-logical arguments under belief and logic instructions across training blocks of Experiment 2. The top row summarises the results of the excluded model. The bottom row shows the results of the full model.

Figure S2 also depicts the distributions of (pseudo-) validity effects under experimental conditions of Experiment 2 for both models. As you can see in these figures, the pattern of the results after excluding participants remained highly similar to the main analysis with all participants included.


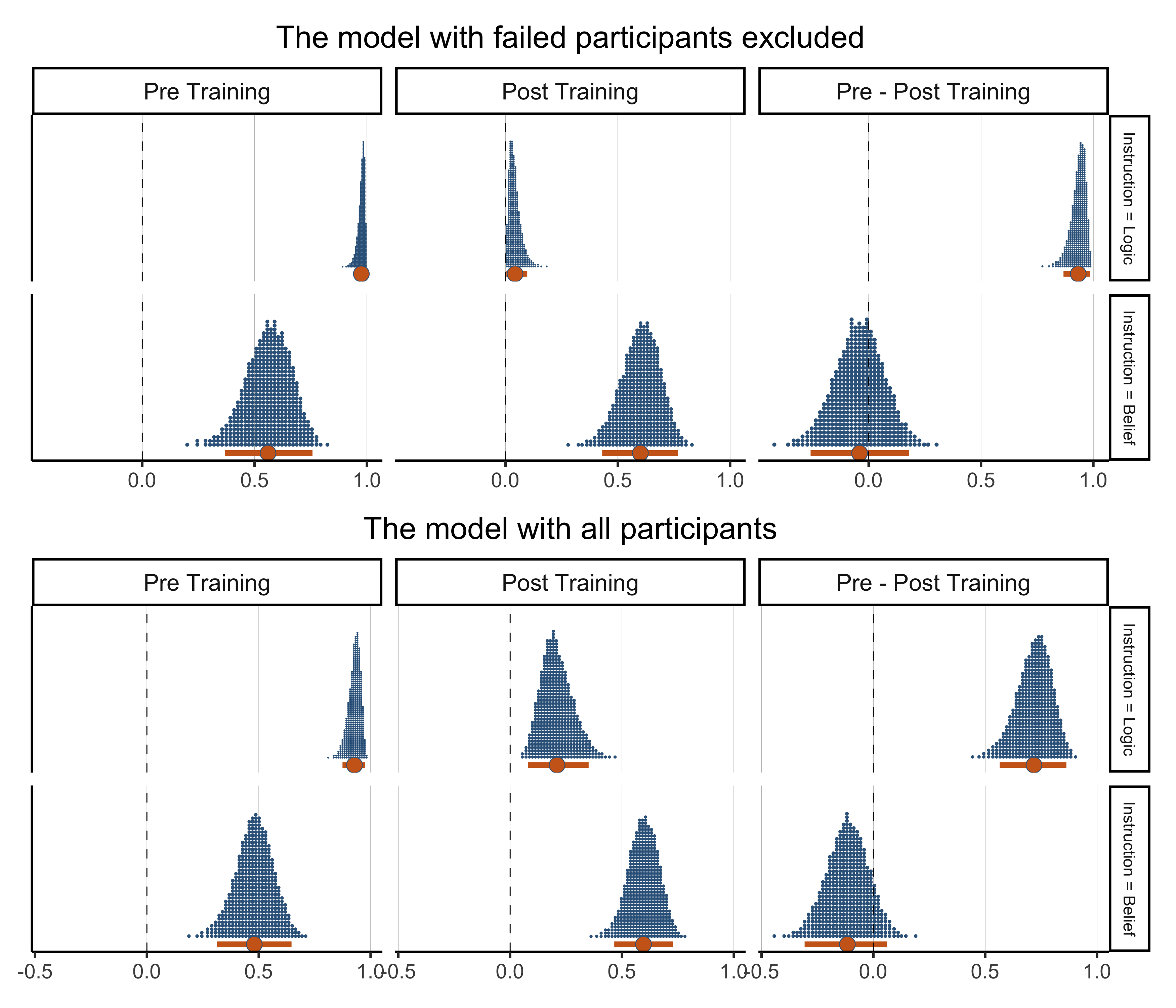


Figure S2: The distributions of (pseudo-) validity effects under logic and belief instructions of Experiment 2 in each training block along with their contrast. The top row summarises the results of the excluded model. The bottom row shows the results of the full model.

## 4.2 Model Specifications

To see the model structures and prior specifications, click on the “Code” button below.

#-------------------------------------------------------------------------------#
#----------------------------- Experiment 1 ----------------------------#
#-------------------------------------------------------------------------------#

# Logic Judgments
m1_logic_exp1 <- brm(response ~ pseudo_logic*belief*argument_type*block +
 (pseudo_logic*belief*argument_type*block|subject)+
 (pseudo_logic*belief*argument_type*block|content),
 data=data_exp1_logic,
 family=bernoulli("logit"),
 prior=c(prior(normal(0, 2), class = Intercept),
 prior(normal(0, 2), class = b),
 prior(cauchy(0, 1), class = sd),
 prior(lkj(2), class = cor)),
 sample_prior = TRUE,
 cores = 4, warmup = 1000, iter = 5000, chains = 4, seed = 123,
 save_all_pars=TRUE,
 file = here("Output","brms_fits","Exp1_logic_brms_fit1")
)


# Belief Judgments
m1_belief_exp1 <- brm(response ~ pseudo_logic*belief*argument_type*block +
 (pseudo_logic*belief*argument_type*block|subject)+
 (pseudo_logic*belief*argument_type*block|content),
 data=data_exp1_belief,
 family=bernoulli("logit"),
 prior=c(prior(normal(0, 2), class = Intercept),
 prior(normal(0, 2), class = b),
 prior(cauchy(0, 1), class = sd),
 prior(lkj(2), class = cor)),
 sample_prior = TRUE,
 cores = 4, warmup = 1000, iter = 5000, chains = 4, seed = 123,
 save_all_pars=TRUE,
 file = here("Output","brms_fits","Exp1_belief_brms_fit1")
)


#-------------------------------------------------------------------------------#
#-------------------------------- Experiment 2 -------------------------#
#-------------------------------------------------------------------------------#

# Logic Judgments
m1_logic_exp2 <- brm(response ~ pseudo_logic*belief*argument_type*block +
 (pseudo_logic*belief*argument_type*block|subject)+
 (pseudo_logic*belief*argument_type*block|content),
 data=data_exp2_logic,
 family=bernoulli("logit"),
 prior=c(prior(normal(0, 2), class = Intercept),
 prior(normal(0, 2), class = b),
 prior(cauchy(0, 1), class = sd),
 prior(lkj(2), class = cor)),
 sample_prior = TRUE,
 cores = 4, warmup = 1000, iter = 5000, chains = 4, seed = 123,
 save_all_pars=TRUE,
 file = here("Output","brms_fits","Exp2_logic_brms_fit1")
)


# Belief Judgments
m1_belief_exp2 <- brm(response ~ pseudo_logic*belief*argument_type*block +
 (pseudo_logic*belief*argument_type*block|subject)+
 (pseudo_logic*belief*argument_type*block|content),
 data=data_exp2_belief,
 family=bernoulli("logit"),
 prior=c(prior(normal(0, 2), class = Intercept),
 prior(normal(0, 2), class = b),
 prior(cauchy(0, 1), class = sd),
 prior(lkj(2), class = cor)),
 sample_prior = TRUE,
 cores = 4, warmup = 1000, iter = 5000, chains = 4, seed = 123,
 save_all_pars=TRUE,
 file = here("Output","brms_fits","Exp2_belief_brms_fit1")
)

##

## 4.3 Model Outputs

Table S3: The outputs of the Bayesian model for logic judgments in Experiment 1

| **Term** | **Estimate** | **SD** | **CI Low** | **CI High** |
| --- | --- | --- | --- | --- |
| (Intercept) | -0.454 | 0.084 | -0.622 | -0.294 |
| pseudo_logic1 | 2.021 | 0.114 | 1.801 | 2.252 |
| belief1 | 0.820 | 0.090 | 0.647 | 1.000 |
| argument_type1 | -0.012 | 0.053 | -0.119 | 0.089 |
| block1 | 0.277 | 0.062 | 0.158 | 0.401 |
| pseudo_logic1:belief1 | 0.083 | 0.056 | -0.026 | 0.193 |
| pseudo_logic1:argument_type1 | 0.260 | 0.063 | 0.137 | 0.384 |
| belief1:argument_type1 | 0.065 | 0.054 | -0.041 | 0.173 |
| pseudo_logic1:block1 | 0.251 | 0.099 | 0.057 | 0.447 |
| belief1:block1 | 0.160 | 0.070 | 0.025 | 0.302 |
| argument_type1:block1 | -0.089 | 0.054 | -0.193 | 0.016 |
| pseudo_logic1:belief1:argument_type1 | -0.027 | 0.058 | -0.141 | 0.085 |
| pseudo_logic1:belief1:block1 | 0.055 | 0.057 | -0.057 | 0.168 |
| pseudo_logic1:argument_type1:block1 | -0.244 | 0.057 | -0.359 | -0.132 |
| belief1:argument_type1:block1 | 0.028 | 0.055 | -0.080 | 0.139 |
| pseudo_logic1:belief1:argument_type1:block1 | -0.053 | 0.050 | -0.153 | 0.046 |

Table S4: The outputs of the Bayesian model for belief judgments in Experiment 1

| **Term** | **Estimate** | **SD** | **CI Low** | **CI High** |
| --- | --- | --- | --- | --- |
| (Intercept) | 0.097 | 0.082 | -0.066 | 0.261 |
| pseudo_logic1 | 1.053 | 0.097 | 0.865 | 1.249 |
| belief1 | 2.291 | 0.143 | 2.012 | 2.580 |
| argument_type1 | -0.012 | 0.058 | -0.126 | 0.103 |
| block1 | 0.049 | 0.067 | -0.083 | 0.180 |
| pseudo_logic1:belief1 | 0.132 | 0.058 | 0.018 | 0.248 |
| pseudo_logic1:argument_type1 | 0.152 | 0.062 | 0.031 | 0.278 |
| belief1:argument_type1 | 0.095 | 0.062 | -0.028 | 0.219 |
| pseudo_logic1:block1 | -0.016 | 0.075 | -0.166 | 0.131 |
| belief1:block1 | 0.161 | 0.074 | 0.016 | 0.309 |
| argument_type1:block1 | -0.053 | 0.055 | -0.162 | 0.055 |
| pseudo_logic1:belief1:argument_type1 | -0.046 | 0.063 | -0.169 | 0.079 |
| pseudo_logic1:belief1:block1 | 0.098 | 0.059 | -0.017 | 0.213 |
| pseudo_logic1:argument_type1:block1 | -0.132 | 0.053 | -0.240 | -0.029 |
| belief1:argument_type1:block1 | -0.066 | 0.058 | -0.183 | 0.047 |
| pseudo_logic1:belief1:argument_type1:block1 | -0.050 | 0.063 | -0.176 | 0.072 |

Table S5: The outputs of the Bayesian model for logic judgments in Experiment 2

| **Term** | **Estimate** | **SD** | **CI Low** | **CI High** |
| --- | --- | --- | --- | --- |
| (Intercept) | -0.887 | 0.207 | -1.311 | -0.497 |
| pseudo_logic1 | 3.779 | 0.312 | 3.209 | 4.425 |
| belief1 | 1.118 | 0.203 | 0.734 | 1.532 |
| argument_type1 | 0.785 | 0.165 | 0.472 | 1.123 |
| block1 | 0.820 | 0.179 | 0.489 | 1.195 |
| pseudo_logic1:belief1 | -0.057 | 0.156 | -0.373 | 0.237 |
| pseudo_logic1:argument_type1 | 1.190 | 0.191 | 0.839 | 1.587 |
| belief1:argument_type1 | 0.110 | 0.140 | -0.152 | 0.402 |
| pseudo_logic1:block1 | 0.189 | 0.199 | -0.196 | 0.583 |
| belief1:block1 | 0.235 | 0.152 | -0.066 | 0.533 |
| argument_type1:block1 | -0.588 | 0.166 | -0.931 | -0.273 |
| pseudo_logic1:belief1:argument_type1 | 0.017 | 0.113 | -0.203 | 0.245 |
| pseudo_logic1:belief1:block1 | 0.207 | 0.131 | -0.044 | 0.469 |
| pseudo_logic1:argument_type1:block1 | -0.642 | 0.171 | -0.991 | -0.317 |
| belief1:argument_type1:block1 | -0.018 | 0.118 | -0.254 | 0.215 |
| pseudo_logic1:belief1:argument_type1:block1 | -0.103 | 0.109 | -0.319 | 0.109 |

Table S6: The outputs of the Bayesian model for belief judgments in Experiment 1

| **Term** | **Estimate** | **SD** | **CI Low** | **CI High** |
| --- | --- | --- | --- | --- |
| (Intercept) | 0.306 | 0.129 | 0.055 | 0.563 |
| pseudo_logic1 | 1.599 | 0.178 | 1.266 | 1.958 |
| belief1 | 2.705 | 0.221 | 2.292 | 3.157 |
| argument_type1 | 0.195 | 0.091 | 0.020 | 0.376 |
| block1 | 0.053 | 0.110 | -0.166 | 0.265 |
| pseudo_logic1:belief1 | 0.300 | 0.114 | 0.079 | 0.531 |
| pseudo_logic1:argument_type1 | 0.351 | 0.103 | 0.154 | 0.557 |
| belief1:argument_type1 | 0.292 | 0.101 | 0.102 | 0.503 |
| pseudo_logic1:block1 | -0.136 | 0.118 | -0.372 | 0.094 |
| belief1:block1 | 0.085 | 0.117 | -0.144 | 0.317 |
| argument_type1:block1 | -0.166 | 0.094 | -0.351 | 0.020 |
| pseudo_logic1:belief1:argument_type1 | 0.055 | 0.092 | -0.124 | 0.245 |
| pseudo_logic1:belief1:block1 | 0.185 | 0.100 | -0.014 | 0.382 |
| pseudo_logic1:argument_type1:block1 | 0.019 | 0.096 | -0.169 | 0.211 |
| belief1:argument_type1:block1 | -0.080 | 0.106 | -0.290 | 0.129 |
| pseudo_logic1:belief1:argument_type1:block1 | -0.007 | 0.084 | -0.174 | 0.156 |

## 4.4 Posterior Predictive Checks

As figure S3 shows, the simulated data from our Bayesian models mirrored the original data very well.


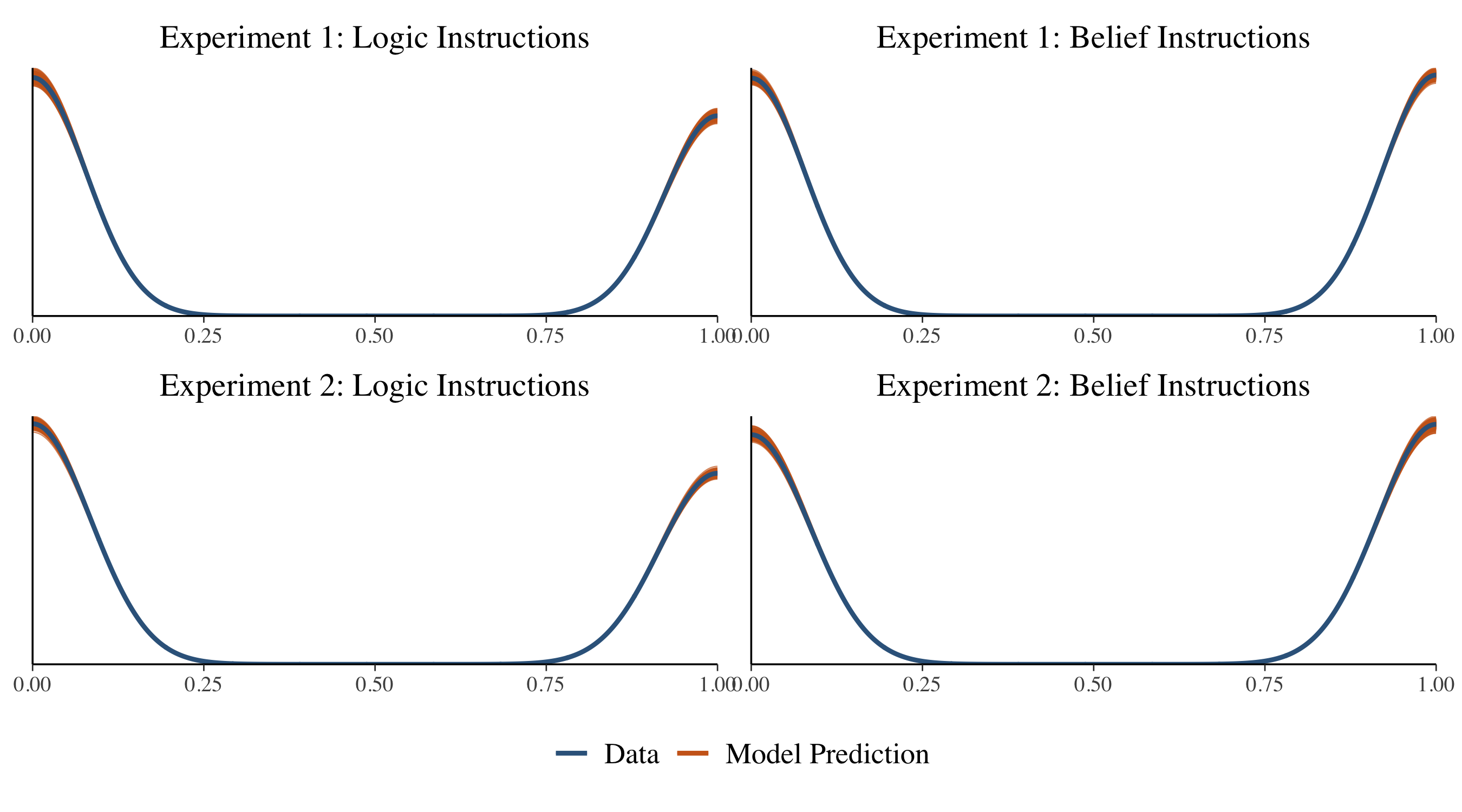


Figure S3: Posterior predictive checks of our Bayesian mixed models. For each plot, we used 500 simulations.

## 4.5 Trace Plots

Figure S4 shows the trace plots for the theoretically significant effects for each model. As the figure shows, the chains have successfully converged. Moreover, none of population-level effects had $\hat{R}$ values larger than 1 which is evidence of convergence.


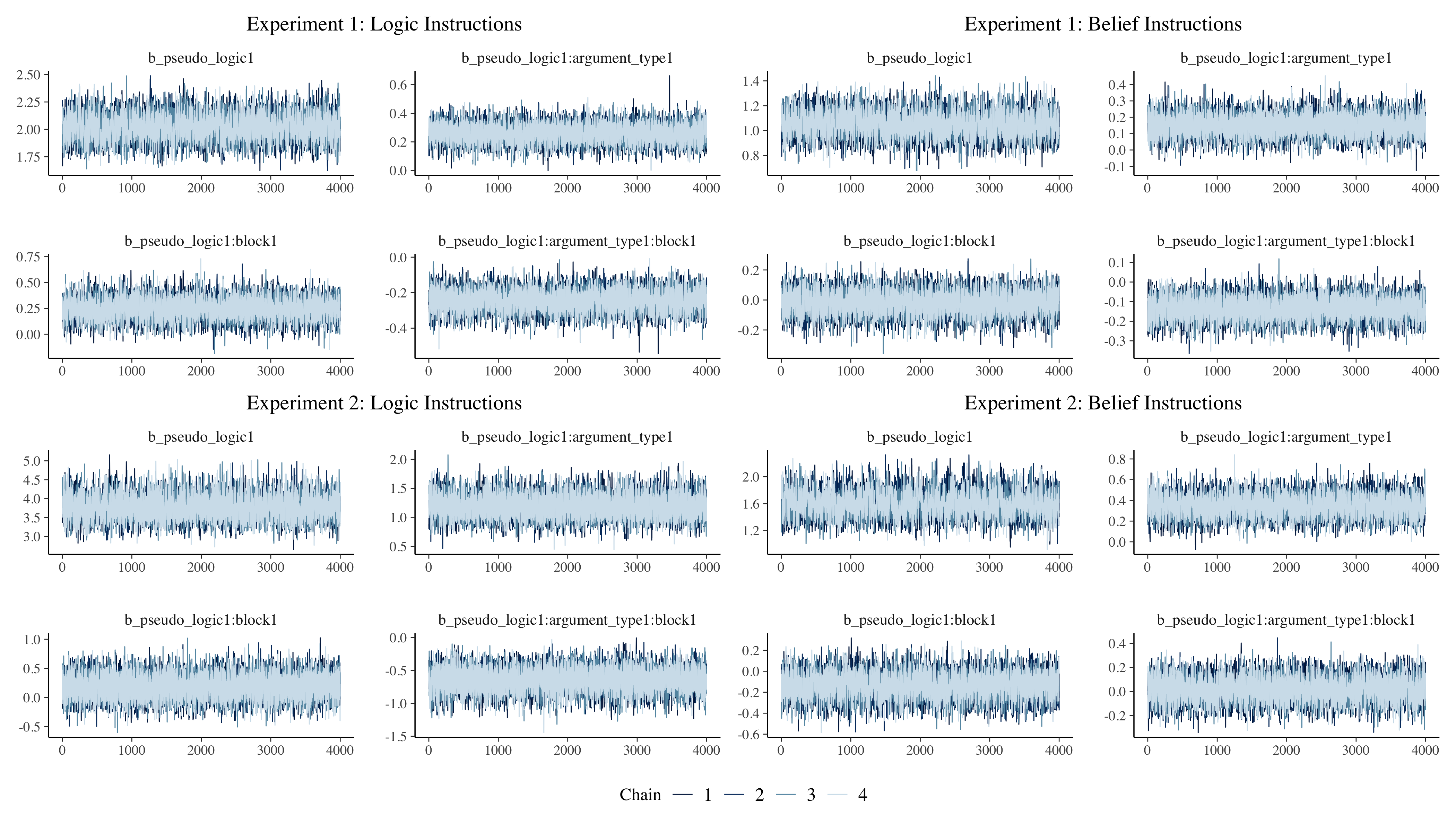


Figure S4: trace plots of the theoretically important terms in each model

## 4.6 Model Comparison

Figure S5 shows the predictive accuracy (i.e., elpd loo) of models with and without the (pseudo-) validity by argument type by block interaction.


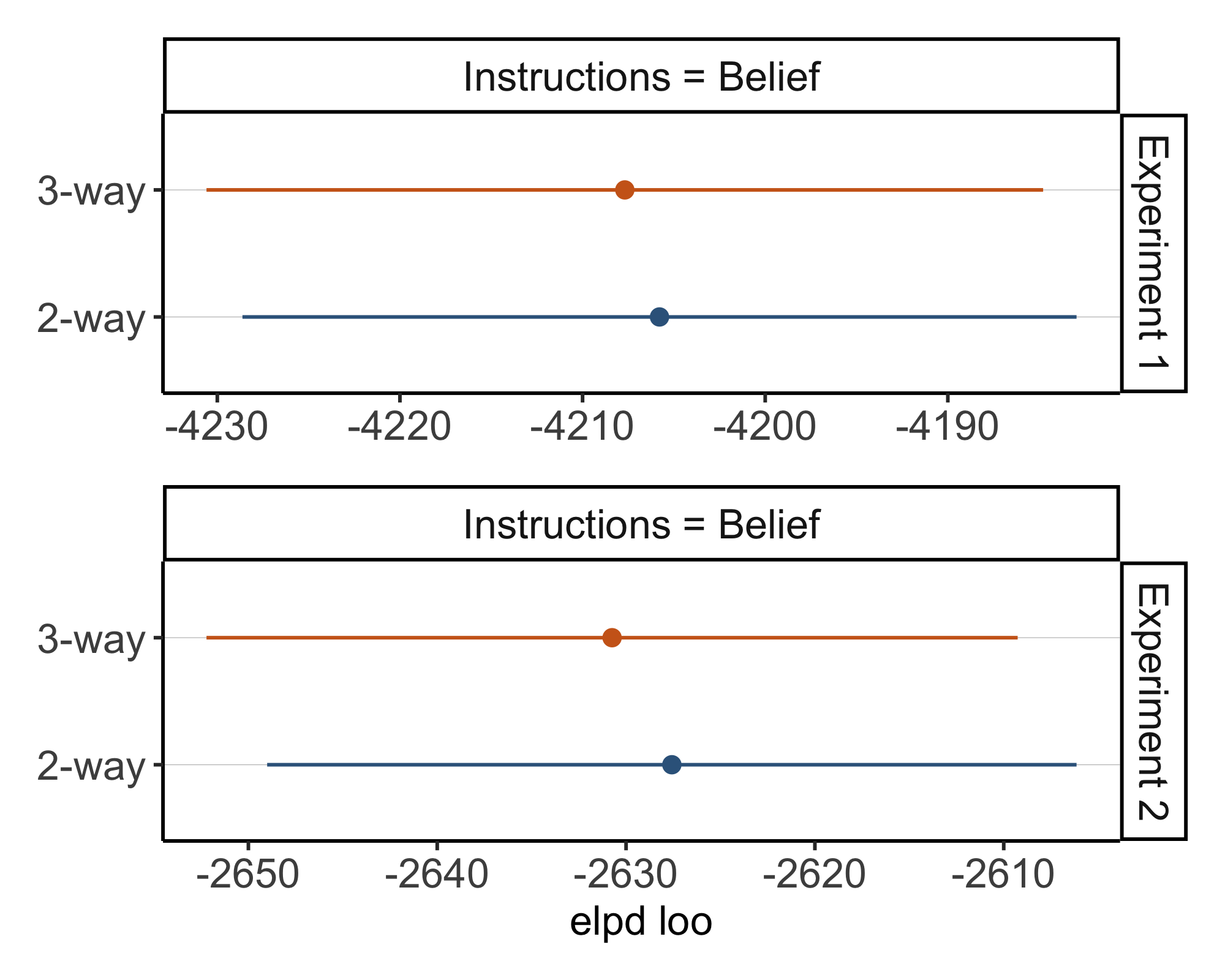


Figure S5: elpd loo of models with and without the 3-way interaction under belief instructions in Experiments 1 and 2. Error bars represents standard errors.

# 5 Further Analyses

## 5.1 Task-Switch Hypothesis

In our three experiments, we utilized a within-subject design, using logic (Task 1) and belief (Task 2) instructions, and observed that the presence of (pseudo-)validity effect in belief judgments. However, this interference could be a result of task-switching rather than the influence of logical or pseudo-logical structures. Specifically, when participants were cued with a different response in switch trials (trials that participants are cued with a response different than the previous trial) compared to repeat trials (trials that cues a same response), a carry-over activation could cause interference and lead to the observed conflict effect. Previous studies have also reported interference effects with a between-subject or block design (Handley et al., 2011, Experiment 1; Pennycook et al., 2013, Experiment 3; Howarth et al., 2016, Experiment 3).

To investigate this further, we analyzed the switch and repeat trials under belief instructions. Our results showed that the (pseudo-)validity effect was not influenced by this factor, as indicated by a non-significant interaction effects (*ps* >.28).

Table S7: The outputs of the mixed model with (pseudo-) validity, task, and instructions as independent variables and endorsement ratings as dependent variable for aggregated data.

| **Effect** | **df** | **F** | **p.value** |
| --- | --- | --- | --- |
| pseudo_logic | 1, 20582.21 | 4143.28 *** | <.001 |
| task | 1, 20801.29 | 2.94 + | .086 |
| instruction | 1, 20581.84 | 82.21 *** | <.001 |
| pseudo_logic:task | 1, 20821.65 | 0.74 | .388 |
| pseudo_logic:instruction | 1, 20582.08 | 551.89 *** | <.001 |
| task:instruction | 1, 20581.80 | 0.40 | .527 |
| pseudo_logic:task:instruction | 1, 20823.48 | 1.19 | .276 |

This suggests that the (pseudo-) validity effect may not appear due to task-switching, but also had a basis in the (pseudo-) intuitive logic explanation. Figure S5 provides a clear illustration of the (pseudo-) validity effect on both switch and repeat trials.


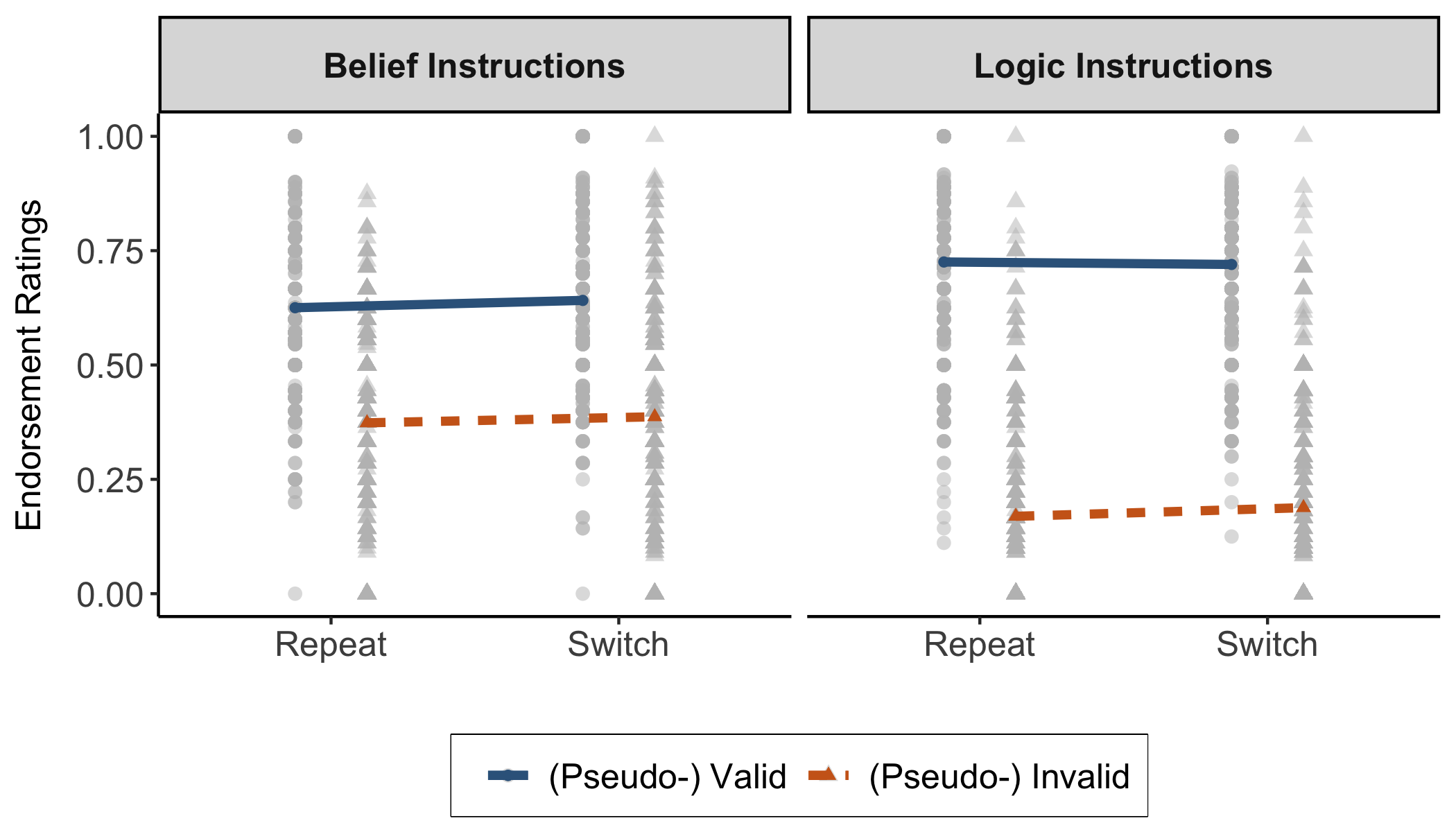


Figure S5: the (pseudo-) validity effects under switch and repeat trials of logic and belief instructions

## 5.2 Biconditional Reading of Conditionals

In this section, we compared the endorsement ratings of “MP” and “AC” arguments under logic instructions. The more marked (pseudo-)validity effect on MP arguments challenges the biconditional account of our results (see figure below). Our results are consistent with evidence in the literature against a routine biconditional interpretation of conditional assertions.

Table S8: The outputs of the mixed model with (pseudo-) validity and argument subtype (MP vs. AC) as independent variables and endorsement ratings as dependent variable for aggregated data.

| **Effect** | **df** | **F** | **p.value** |
| --- | --- | --- | --- |
| pseudo_logic | 1, 7101.82 | 4179.40 *** | <.001 |
| sub_type | 1, 7101.82 | 108.01 *** | <.001 |
| pseudo_logic:sub_type | 1, 7101.82 | 210.31 *** | <.001 |


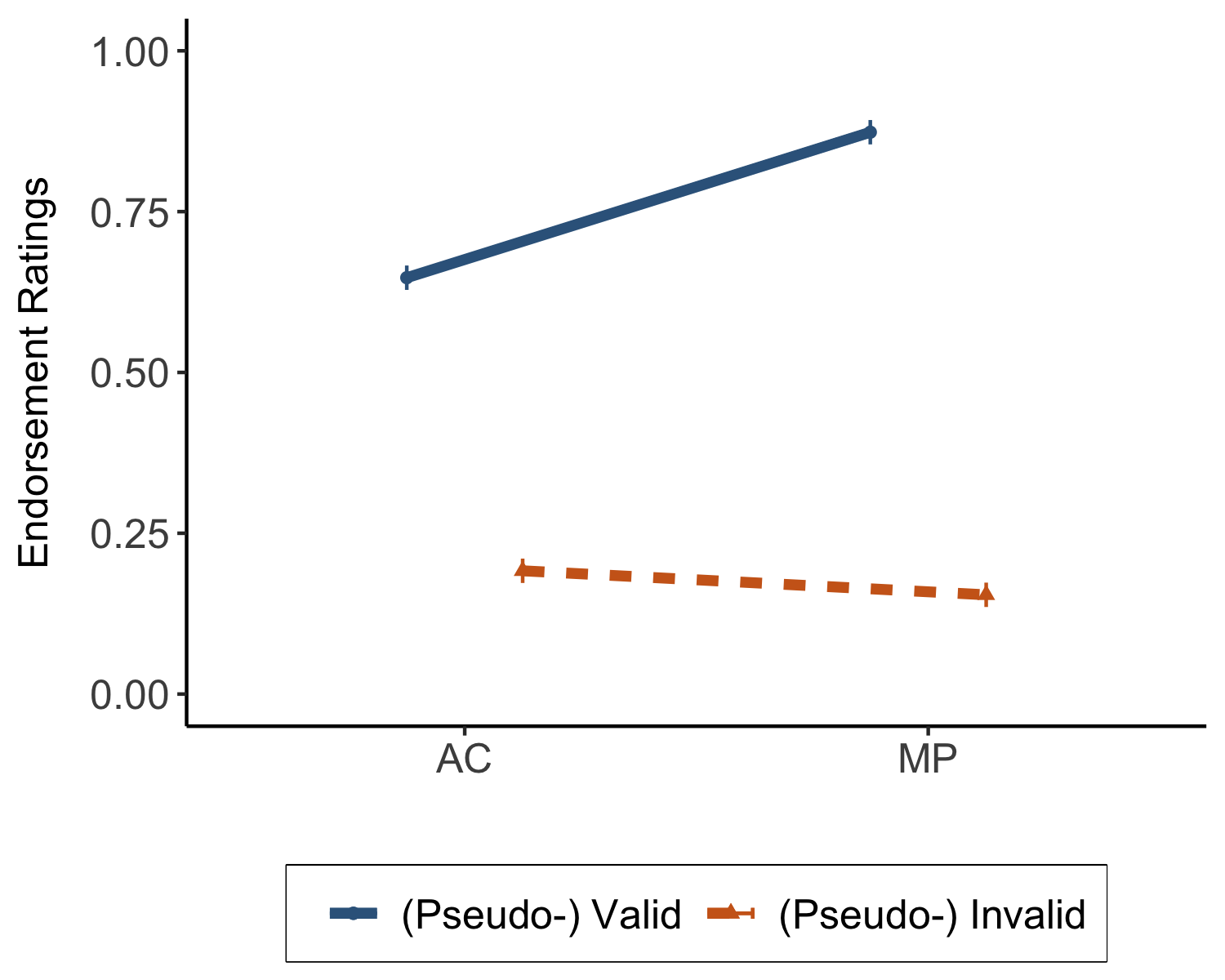


Figure S6: the (pseudo-) validity effects on MP and AC arguments under logic instructions

## 5.3 Descriptive Results

The figures below shows endorsement ratings for all experimental factors of Experiments 1 and 2.


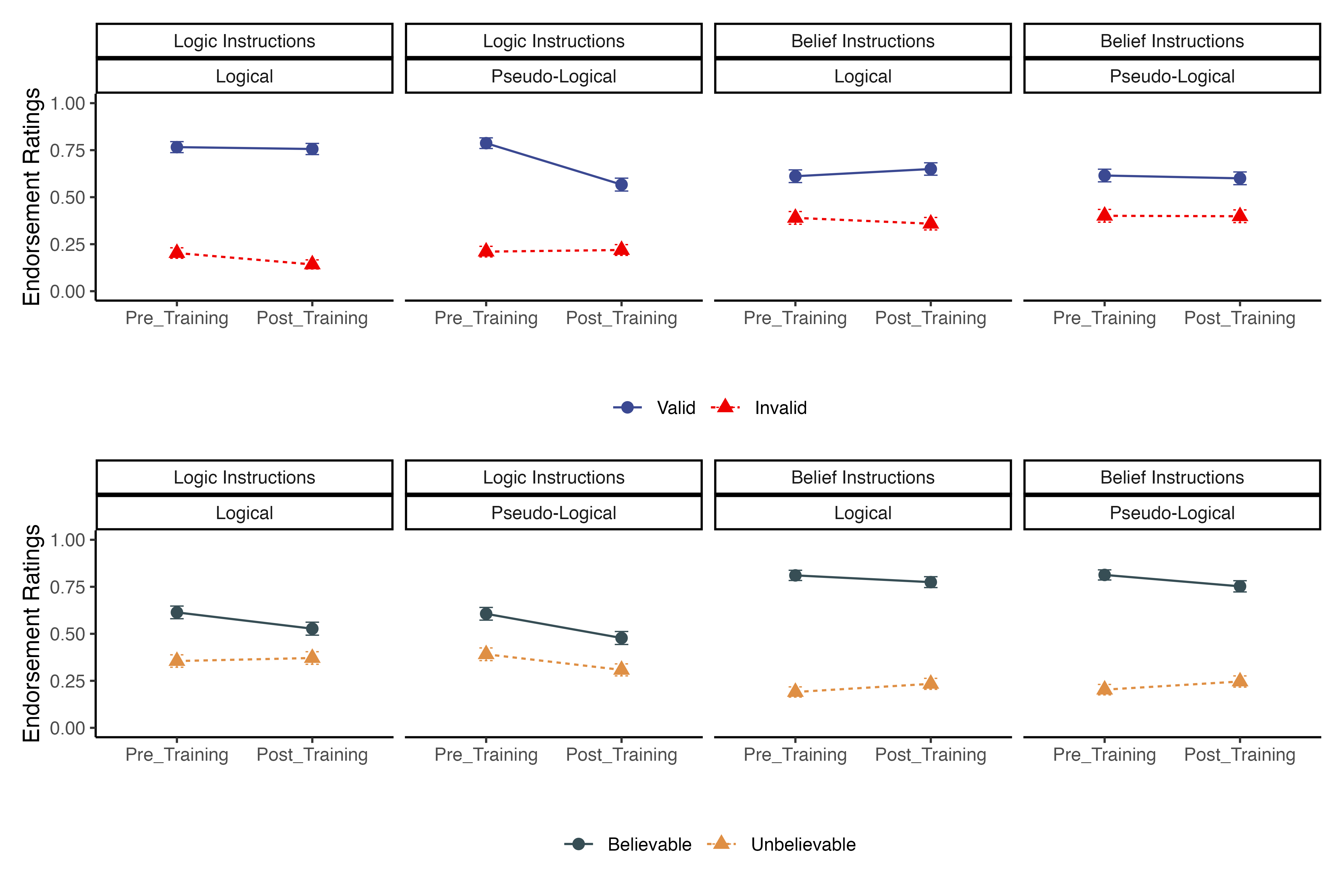


Figure S7: Endorsement ratings of (pseudo) validity and believability conditions on logical and pseudo-logical arguments under belief and logic instructions across training blocks of Experiment 1. Error bars represent 95% CI.


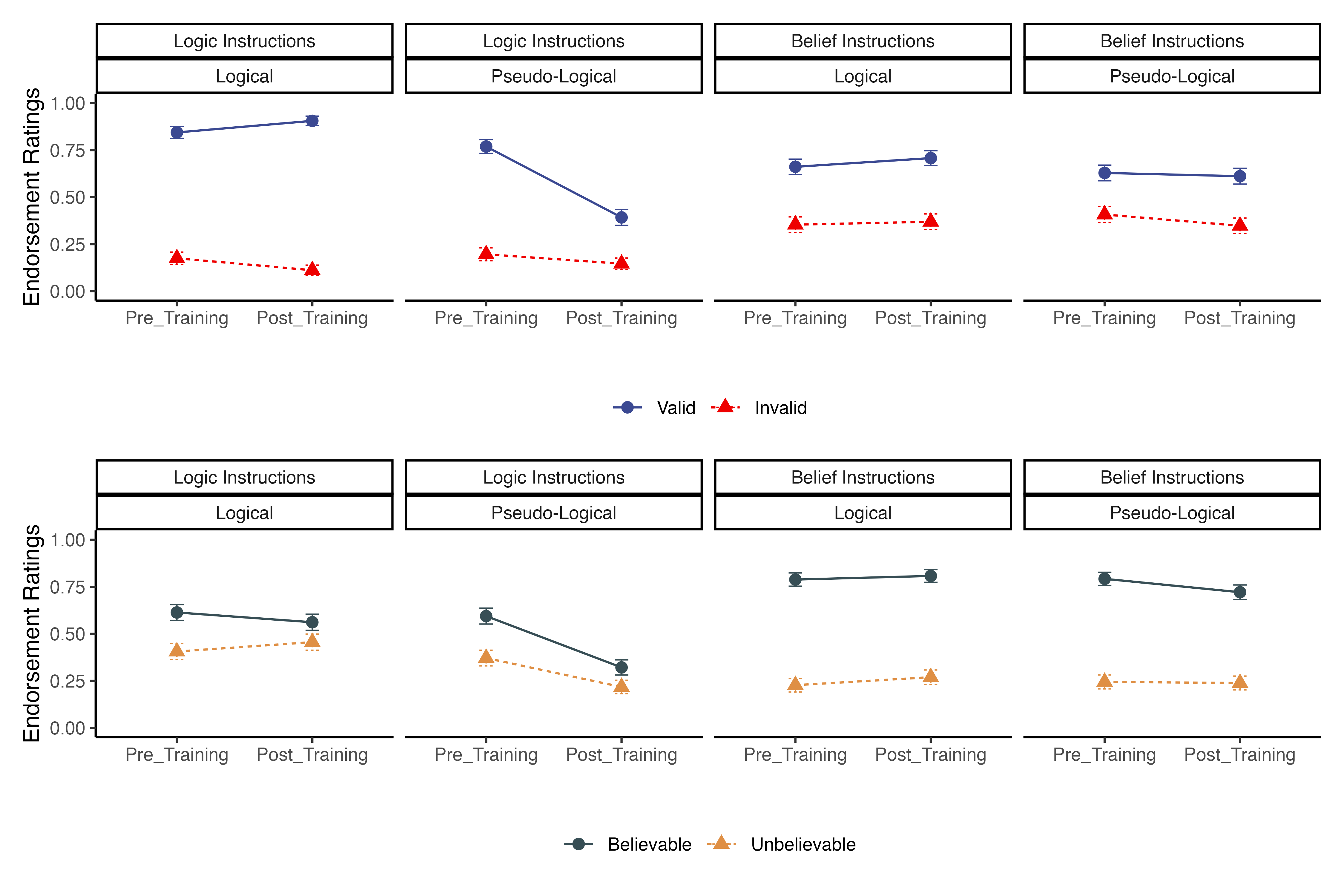


Figure S8: Endorsement ratings of (pseudo) validity and believability conditions on logical and pseudo-logical arguments under belief and logic instructions across training blocks of Experiment 2. Error bars represent 95% CI.

## 5.4 Individual Differences

We examined how endorsement of valid vs. invalid arguments differed across Pseudo-Logical and Logical arguments in two experiments. For each participant, we computed the difference in endorsement rates (Valid - Invalid) and plotted the distribution separately for Pre-training and Post-training blocks under Logic and Belief instructions. These distributions illustrate how participants’ reasoning changed with training.

Next, to compare the logic-belief effect across argument types, we calculated the difference in the logic-belief effect (i.e., valid - invalid) under belief instructions between Pseudo-Logical and Logical arguments. This highlights whether participants showed a stronger validity effect for one argument type over the other. A vertical reference line at zero indicates no difference, helping to interpret the direction of these effects.


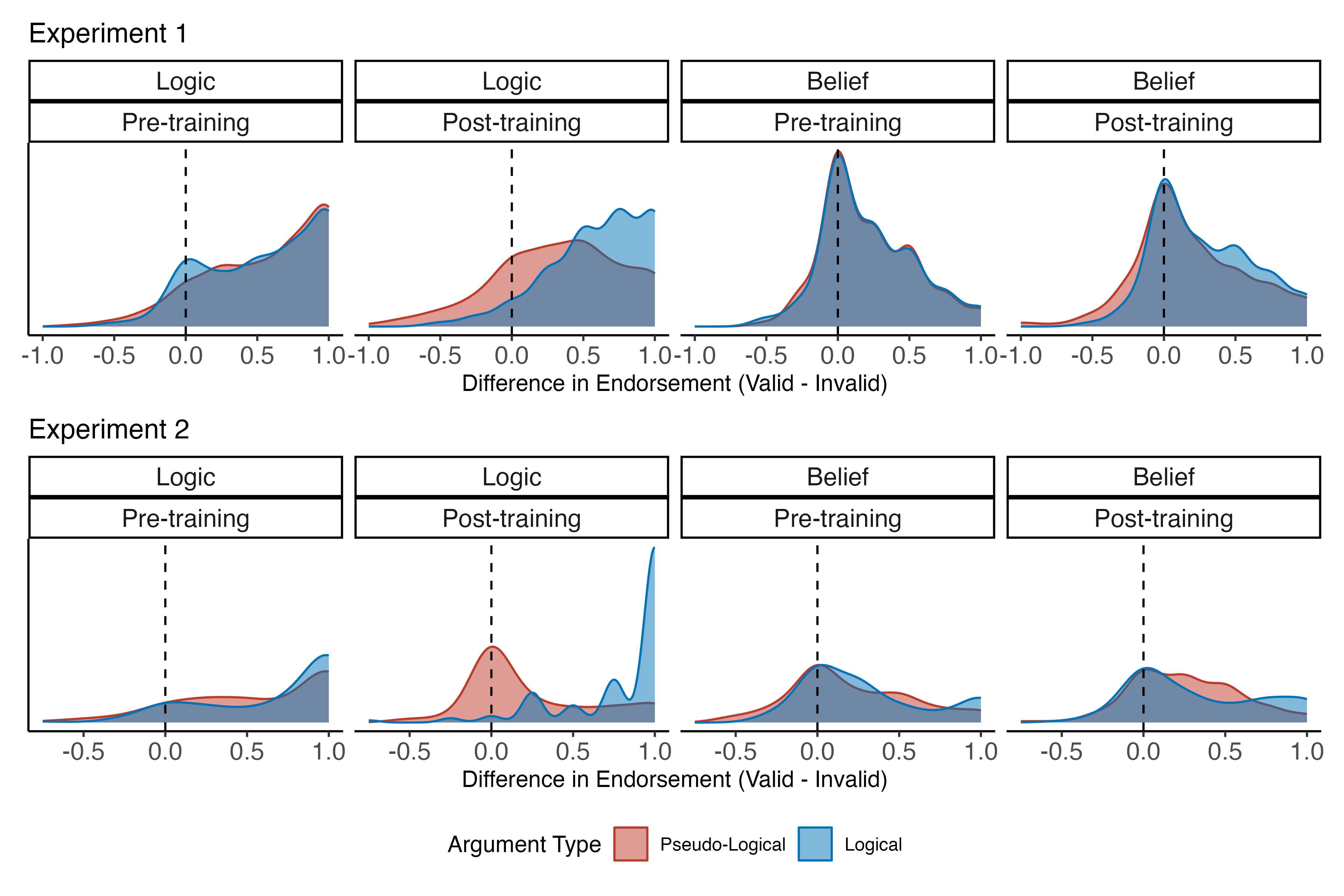


Figure S9: The distribution of the logic-belief effect (i.e., endorsement of valid vs. invalid arguments) across training blocks, instructions, and argument type in two experiments.


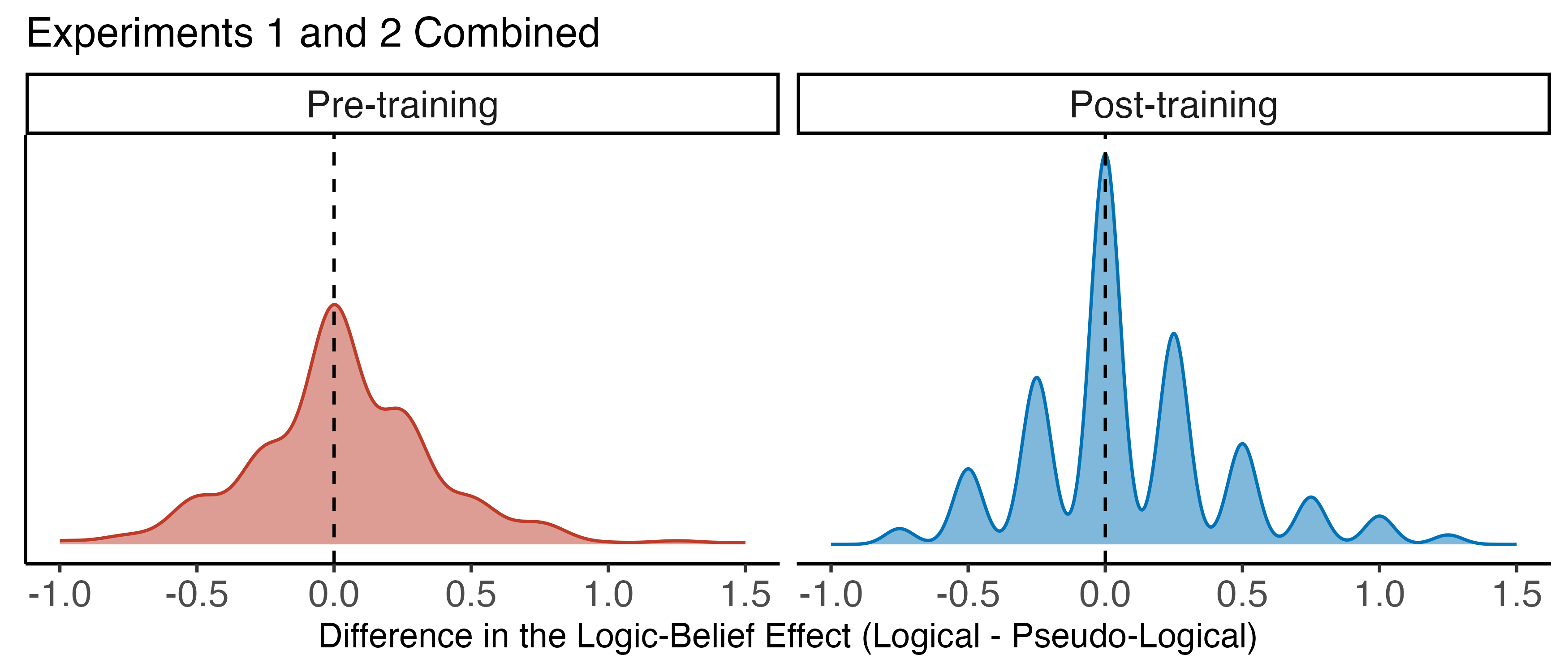


Figure S10: The distribution of the logic-belief effect (i.e., endorsement of valid vs. invalid arguments) under belief instructions across Pseudo-Logical and Logical arguments in two experiments.

## 5.5 Matching vs. Logic

To examine how logical validity and matching conditions influence endorsement rates, we analyzed responses across different training blocks and instructional conditions. Specifically, we compared the endorsement rates of arguments that were either aligned (e.g., Valid-Match) or misaligned (e.g., Invalid-Non-Match) in their logical structure and matching condition. This analysis was conducted separately for Experiment 1 and Experiment 2.

For each dataset, we classified arguments based on their logical validity (Valid vs. Invalid) and matching condition (Match vs. Non-Match), creating three key categories: Valid-Match, Invalid-Match, and Invalid-Non-Match. We computed mean endorsement rates and their associated confidence intervals for each condition, separately for the pre-training and post-training blocks, as well as for belief and logic instructional conditions.


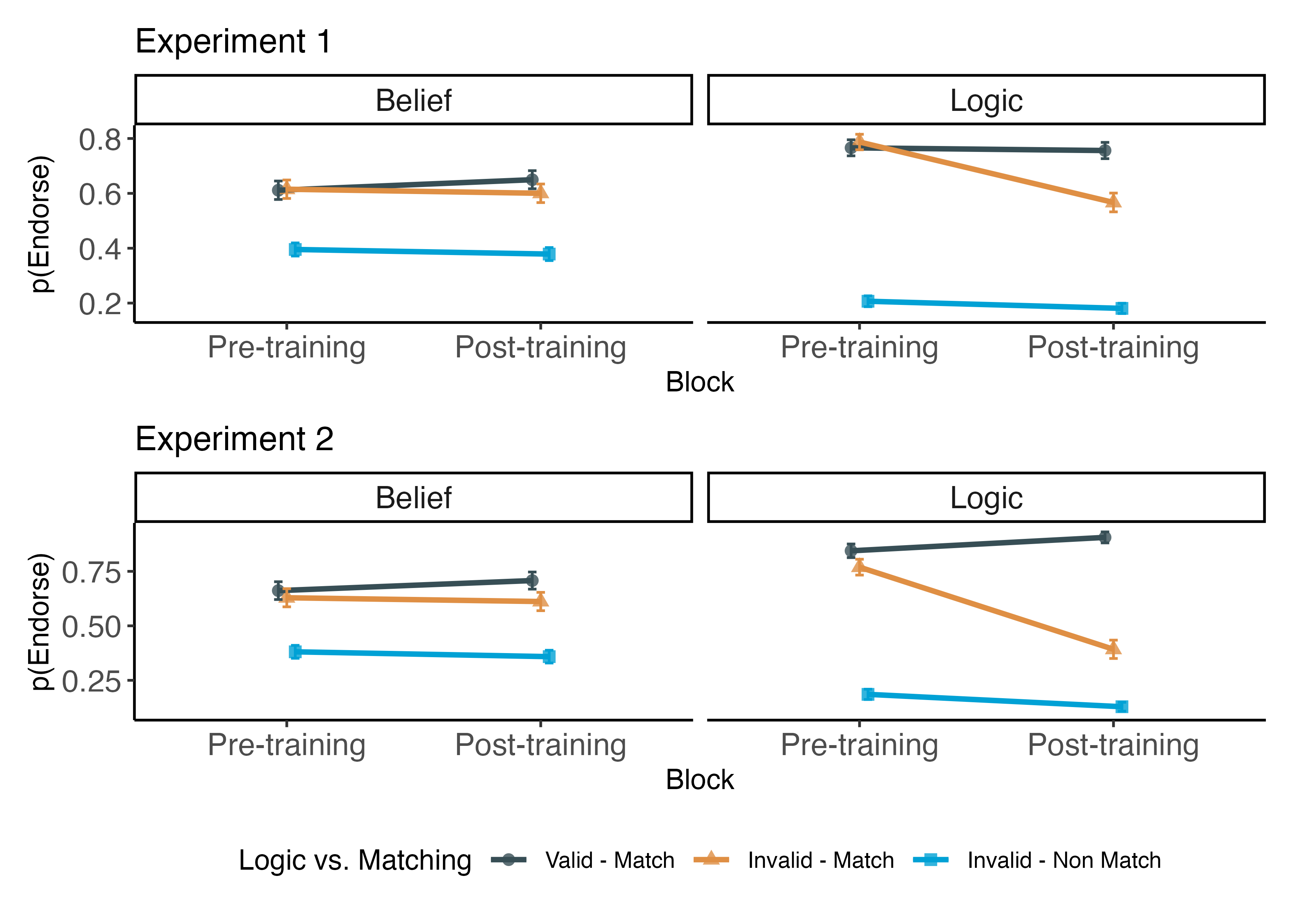


Figure S11: Endorsement rates for arguments categorized by logical validity and matching condition across pre-training and post-training blocks under logic and belief instrcutions of Experiments 1 (top panel) and 2 (botoom panel). Error bars represent 95% confidence intervals.

The results showed that under belief instructions, participants endorsed valid-match and invalid-match arguments at similar rates, and training had little effect in differentiating these conditions. This suggests that individuals relied primarily on matching cues, endorsing matched arguments regardless of logical validity. In contrast, invalid-non-match arguments were mostly rejected. Under logic instructions, a similar pattern emerged, but after training, participants were significantly less likely to endorse invalid-match arguments, indicating that training reduced reliance on the matching heuristic.

To further examine the effects of logical validity, matching condition, and belief on endorsement rates, we conducted linear regression analyses separately for each experiment and training block under belief-based instructions. The models included logic (Valid vs. Invalid), matching (Match vs. Non-Match), and belief (Believable vs. Unbelievable) as predictors of endorsement responses. Figure below presents the estimated regression coefficients for each predictor, with separate panels for Experiments 1 and 2.


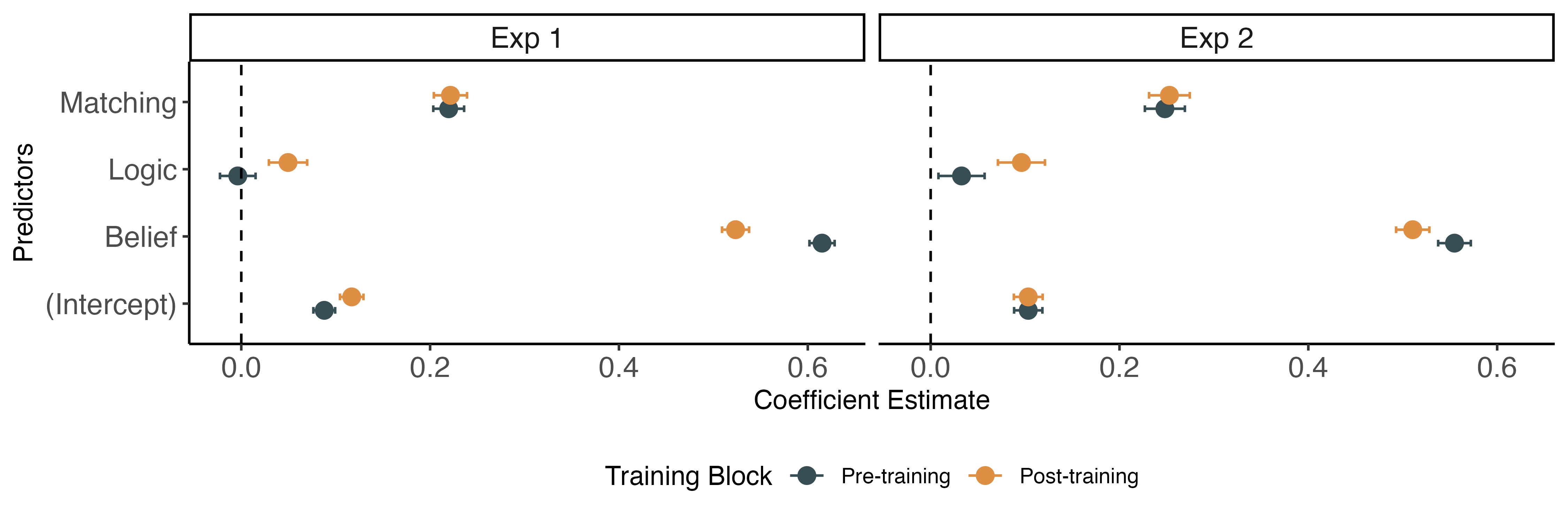


Figure S12: Estimated regression coefficients for the effects of logic, matching, and belief on endorsement rates under belief-based instructions, separately for pre-training and post-training blocks. The x-axis represents the predictors included in the regression models (Logic, Matching, and Belief), while the y-axis indicates the coefficient estimates. Points represent estimated coefficients, with error bars reflecting standard errors. Dashed horizontal lines indicate zero-effect baselines. Separate panels show results for Experiment 1 and Experiment 2, while training blocks (Pre-training vs. Post-training) are distinguished by color.

The results revealed that belief had the strongest effect on endorsement rates, with believable arguments receiving significantly higher endorsements than unbelievable ones. Matching also played a significant role, with matched arguments endorsed more frequently than non-matching ones. However, logical validity (Valid vs. Invalid) had little to no impact on endorsement rates, further supporting the idea that belief-driven reasoning dominates over logical structure. The effects of matching and belief persisted after training, though training effects varied between experiments.
